# Behavioural traits propagate across generations via segregated iterative-somatic and gametic epigenetic mechanisms

**DOI:** 10.1038/ncomms11492

**Published:** 2016-05-13

**Authors:** Emma Mitchell, Shifra L. Klein, Kimon V. Argyropoulos, Ali Sharma, Robin B. Chan, Judit Gal Toth, Luendreo Barboza, Charlotte Bavley, Analia Bortolozzi, Qiuying Chen, Bingfang Liu, Joanne Ingenito, Willie Mark, Jarrod Dudakov, Steven Gross, Gilbert Di Paolo, Francesc Artigas, Marcel van den Brink, Miklos Toth

**Affiliations:** 1 Department of Pharmacology, Weill Cornell Medical College, 1300 York Avenue, New York, New York 10065, USA; 2 Department of Immunology, Memorial Sloan Kettering Cancer Center, 1275 York Avenue, New York, New York 10065, USA; 3 Department of Pathology and Cell Biology, Columbia University Medical Center, 630 West 168th Street, New York, New York 10032, USA; 4 Taub Institute for Research on Alzheimer's Disease and the Aging Brain, Columbia University Medical Center, New York, New York 10032, USA; 5 Department of Neurochemistry and Neuropharmacology, Institut d'Investigacions Biomèdiques de Barcelona (IIBB-CSIC), Rosselló, 161, Barcelona 08036, Spain; 6 Centro de Investigación Biomédica en Red de Salud Mental (CIBERSAM), Barcelona 08036, Spain; 7 Institut d'Investigacions Biomèdiques August Pi i Sunyer (IDIBAPS), Barcelona 08036, Spain; 8 Mouse Genetics Core Facility, Sloan Kettering Institute, Memorial Sloan Kettering Cancer Center, 417 East 68th Street, New York, New York 10065, USA

## Abstract

Parental behavioural traits can be transmitted by non-genetic mechanisms to the offspring. Although trait transmission via sperm has been extensively researched, epidemiological studies indicate the exclusive/prominent maternal transmission of many non-genetic traits. Since maternal conditions impact the offspring during gametogenesis and through fetal/early-postnatal life, the resultant phenotype is likely the aggregate of consecutive germline and somatic effects; a concept that has not been previously studied. Here, we dissected a complex maternally transmitted phenotype, reminiscent of comorbid generalized anxiety/depression, to elementary behaviours/domains and their transmission mechanisms in mice. We show that four anxiety/stress-reactive traits are transmitted via independent iterative-somatic and gametic epigenetic mechanisms across multiple generations. Somatic/gametic transmission alters DNA methylation at enhancers within synaptic genes whose functions can be linked to the behavioural traits. Traits have generation-dependent penetrance and sex specificity resulting in pleiotropy. A transmission-pathway-based concept can refine current inheritance models of psychiatric diseases and facilitate the development of better animal models and new therapeutic approaches.

## Abstract (web summary)

Physiological effects of psychological stress and infection in mothers can increase the incidence of anxiety and psychiatric diseases in offsprings and in subsequent generation. Here, Miklos Toth and colleagues show that intergenerational inheritance of neurological traits is propagated across multiple generations independently by parallel non-genetic mechanisms involving independent segregation of epigenetic specific loci.

## Graphical abstract

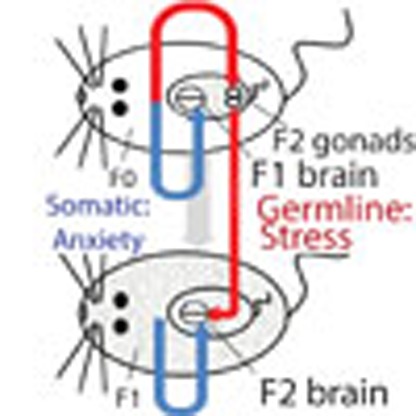

The concept of ‘non-genetic' inheritance of parental traits is gaining acceptance as a significant contributor to the development of disease phenotypes, including psychiatric disorders^1^. For example, stress and resulting stress disorders in parents increase the risk for post traumatic stress disorder, depression and anxiety disorders in their progeny. Many aspects of this ‘intergenerational' transmission paradigm can be reproduced in rodents. In particular, parental stress was shown in several studies to result in abnormal emotional behaviour in the offspring^2^. Some human studies also suggest the transmission of parental behavioural/psychiatric conditions to the grandchildren^3^. As a result, the mechanism of ‘multigenerational' transmission of parental traits has been extensively studied in rodent models, especially through the male line because of its relatively straightforward interpretation via germ cells and the ease of obtaining sperm for epigenetic studies^4^.

However, epidemiological studies indicate that many inter/multigenerational non-genetic behavioural phenotypes are prominently or exclusively transmitted through the maternal line^1^,^5^. This is not surprising because, in contrast to paternal, maternal conditions can impact the offspring during gametogenesis and through fetal life, increasing phenotypic complexity and the overall inter/multigenerational effect. One prominent example is the increased vulnerability of adult children and grandchildren of Holocaust survivors to psychological distress^3^,^6^. Additional studies suggest that maternal stress and infection increase the incidence of anxiety, depression, schizophrenia, autism and attention-deficit hyperactivity disorder in the progeny^1^. Non-genetic inheritance can also be initiated by maternal mutations that perturb fetal development, but are not transmitted genetically to the offspring. A recent example relevant to psychiatric conditions is maternal (but not paternal) mutations in tryptophan hydroxylase I (an enzyme responsible for serotonin synthesis in the periphery) resulting in increased risk for attention-deficit hyperactivity disorder in the offspring^7^. Non-genetic multigenerational transmission of behaviour through the female line has also been demonstrated in rodents^8^,^9^.

Although these examples demonstrate the non-genetic transmission of complex behavioural traits via the maternal line across at least two generations and underscore its potential clinical importance, the idea that multifaceted offspring phenotypes can be the aggregate of the consecutive actions of germline and various ‘somatic' maternal effects has not been previously studied as a collective basis for complex diseases. Maternal intergenerational effects during pre/postnatal life are believed to be primarily mediated by hormonal and/or cytokine signalling pathways, emanating from the mother and altering the development of the fetal or neonatal brain^10^,^11^. However, these somatic mechanisms are limited to first-generation phenotypes, unless the maternal phenotype is self-perpetuating, a possibility that has not been explored comprehensively, presumably because its substantiation requires relatively complex embryo transfer and crossfostering experiments. Therefore, the question remains whether multigenerational transmission via the maternal line is gametic, as was found in a recent animal model^9^, and/or somatic, mediated by an iterative process.

To answer this question, we dissected a composite maternally transmitted behavioural phenotype that resembles in dimensions comorbid general anxiety and depression, to elementary behaviours/circuits and their corresponding transmission mechanisms. Reduced level/binding potential of the serotonin_1A_ receptor (5-HT_1A_R) is associated with anxiety, depression and stress disorders^12^,^13^ and an anxiety-like (that is, increased innate fear) phenotype in mice^14^. We previously reported that 5-HT_1A_R^+/−^ dams not only exhibit anxiety and stress-reactivity traits, but also transmit them non-genetically to their F1 wild-type (WT) offspring^15^. Here we show that the elementary traits of the composite phenotype are propagated beyond the F1 generation up to the F3 generation and that, in contrast to Mendelian inheritance, the maternal traits are not inherited in unison, but rather transmitted by segregated somatic and gametic mechanisms, each with generation-dependent penetrance and sex specificity. We also demonstrate that somatic transmission can be iterative and results in a multigenerational phenotype without the involvement of the gametes. Whether iterative somatic or gametic, the transmission mechanisms converge on enhancer-like sequences within synaptic genes, implicating abnormal neuronal signalling in the manifestation of the offspring phenotype. Our data introduce segregated transmission of non-genetic traits as a mechanism that may explain some aspects of the non-Mendelian propagation of behaviours and dimensions of psychiatric diseases across generations.

## Results

### Non-genetic propagation of behavioural traits

**Figure 1:**
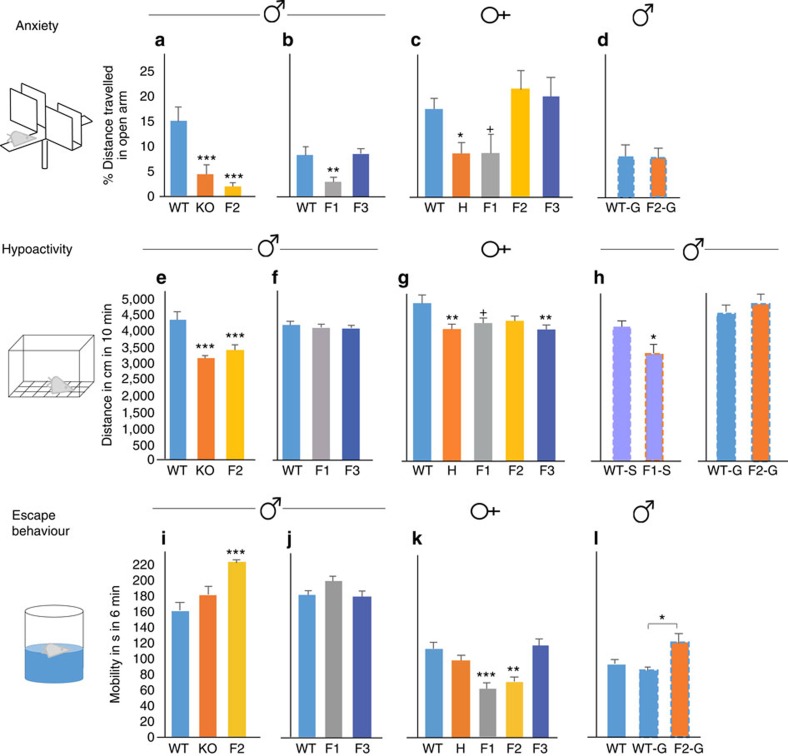
Non-genetic transmission of emotional behavioural traits, associated with maternal 5-HT_1A_R deficit. (**a**) Anxiety-like behaviour in the open arm of the EPM. F2 males, similar to KO males, exhibit increased avoidance (in % of total activity) of the open arm (group-effect in distance travelled; analysis of variance (ANOVA): F_2,35_=13.28, *P*<10^−4^; LSD *post hoc* ***P<0.005 compared with WT; *N*=11, 12 and 15 mice per group). Data presented as mean±s.e.m. All behavioural tests were repeated with three independent cohorts of animals. (**b**) F1, but not F3, males travel less distance in the open arm (group-effect, *F*_2,54_=6.46, *P*=0.003; **P<0.01 compared with WT, *N*=17, 15 and 26 mice). (**c**) Anxiety of female mice (group-effect, F_4,57_=4.22, *P*=0.0046; *N*=12 WT, 13 H, 7 F1, 19 F2 and 11 F3). Significant anxiety in H (*P<0.05) and a trend for anxiety in F1 females (^+^*P* <0.10). (**d**) Males derived by embryo transfer from WT germ cells that were exposed to H (F2-G), as compared with WT (WT-G) grand-maternal environment during gametogenesis exhibit no anxiety-like behaviour (*t*-test:, *T*=−0.202, *P*=0.841, *N*=16 and 20 animals per group). (**e**) Activity, measured as total distance travelled in the open field. F2 males, similar to KO males, exhibit reduced activity (ANOVA: F_2,47_=13.47, *P*<10^−4^; LSD *post hoc* ***P<0.005, *N*=17, 17 and 16). (**f**) Activity of F1 and F3 males is not different from that of WT males. (**g**) Female H and F3 offspring are hypoactive while F1 offspring exhibit a trend for hypoactivity (F_4,49_=3.25, *P*=0.019; **P<0.01, trend ^+^*P*<0.10, *N*=13, 14, 7, 8 and 11). (**h**) Hypoactivity is somatically programmed. WT-S versus F1-S males: *t*-test: *T*=2.286, *P=0.036, *N*=13 and 5 animals per group; WT-G vs. F2-G males: *T*=−1.279, *P*=0.208, *N*=30 and 15. (**i**) Stress reactivity, measured as mobility in the forced swim test. F2 males have increased mobility (ANOVA: F_2,45_=10.12, *P*<0.001; LSD *post hoc* ***P<0.005, *N*=17, 14 and 17). (**j**) Lack of stress phenotype in F1 and F3 males. (**k**) Female F1 and F2 have reduced mobility (F_4,49_=7.92, *P*<10^−4^; **P<0.01, ***P<0.005, *N*=14, 14, 7, 9 and 10). (**l**) Increased stress reactivity of F2 males is gametically programmed (F_2,32_=3.48, *P*=0.042; *P<0.05, *N*=12, 8 and 15).

We reported the propagation of behavioural abnormalities to the genetically WT male offspring of 5-HT_1A_R^+/−^ heterozygote (H) parents and 5-HT_1A_R^−/−^ knockout (KO) surrogate mothers^15^. These behaviours included increased innate fear (anxiety-like behaviour) in the elevated plus maze and increased escape directed behaviour in the forced swim test. Here we tested if these behaviours propagate to the next (F2) generation through the maternal line. F2 males, produced by mating F1 WT females with control WT males (Supplementary Fig. 1), exhibited reduced exploration of the fear-inducing open arm of the elevated plus maze (that is, reduced distance travelled in percent of total distance; Fig. 1a). Total activity was unchanged, indicating that the reduced activity was specific for the open arm and consistent with innate anxiety-like behaviour (Supplementary Fig. 2). As Fig. 1a shows, the anxiety-like phenotype of F2 males were comparable to that of KO males (or H males, not shown^15^), demonstrating the robustness of the non-genetically transmitted behaviour. To test if the anxiety-like behaviour is propagated beyond the F2 generation, F3 males, with age-matched F1, and WT controls were generated. The anxiety-like phenotype was not transmitted to the F3 generation, whereas the F1 males, as in our previous study^15^, exhibited the phenotype (Fig. 1b). Of note, to avoid genetic drift, the H line was backcrossed every five to ten generations to WT mice obtained from large colonies kept at Taconic Biosciences, and then the H and WT lines were re-established; still, non-genetic transmission of the anxiety-like phenotype from H mothers to WT offspring was reproduced following three such backcrosses (H, KO and F1 data from the first and second backcross were reported earlier^15^, whereas the current H, KO, F1, F2 and F3 data were generated with mice from the second and mainly third backcross). Return of the open arm behaviour in F3 generation males to the WT level is also consistent with a non-genetic transmission, as opposed to spurious genetic transmission that would propagate beyond the F2 generation. Next, we generated age-matched WT, H, F1, F2 and F3 females (males were used for epigenetic studies) to study if sex influences transmission. Unlike males, F1 females showed only a trend for increased innate fear and F2 and F3 females were indistinguishable from that of WT (Fig. 1c), indicating sex differences in the transfer and/or manifestation of the anxiety-like phenotype.

We also reported that fetal exposure to the H maternal environment (by WT embryo transfer and consecutive crossfostering to WT mothers) is sufficient to produce increased innate fear in the elevated plus maze in WT mice^15^. We refer to these mice as F1-S, to indicate their ‘somatic' exposure to H mothers. In contrast, anxiety-like behaviour did not result when exposure to the H maternal environment was limited to the germline (G) and early embryogenesis in F1; up to the 2–4 cell stages (Fig. 1d). These F2-G offspring and their WT-G controls were generated by transferring F2 early embryos (exposed at the germ cell stage to the H environment) from F1 donors or WT embryos from WT donors to WT recipients, respectively. Overall, these data are most consistent with a model in which anxiety is transmitted via an iterative non-gametic mechanism from H mothers to the F1 generation, and then from the F1 females to the F2 males.

Although their overall activity was unchanged in the elevated plus maze, F2 males, similar to KO males, exhibited reduced locomotor activity in the larger and less stressful open field test (Fig. 1e). Albeit hypoactivity can be interpreted as a sign of anxiety, in a relatively low stress environment it may rather reflect reduced motivation. In contrast to F2 males, neither F1 nor F3 male offspring showed hypoactivity (Fig. 1f). F1 females showed only a trend for reduced activity, whereas F3, like H females, exhibited significant hypoactivity (Fig. 1g). Overall, these data indicate a phenotype with variable penetrance and sex specificity across three generations. Interestingly, although F1 males showed no phenotype, hypoactivity was robust in F1-S male offspring, indicating that somatic exposure through fetal life to the H maternal environment is sufficient to elicit hypoactivity (Fig. 1h), and that continuous exposure to the H environment during the first 3 weeks of postnatal life in F1 males may moderate the fetal programming effect (Fig. 1f). This could also explain the significant hypoactivity of F2 males (Fig. 1e) because they, like F1-S males, are raised by genetically WT mothers. Non-genetic transmission to grandchildren that skips the F1 generation (but which is propagated through the paternal line) has previously been suggested in human^16^. As we found no apparent change in H maternal care^17^, the moderating effect of the postnatal H environment seems to be non-behavioural and could be mediated by maternal bioactive compound(s) in the milk or by the maternal microbiome. We have previously reported the effect of milk cytokines on the cognitive development in mice^11^, whereas others reported the effect of the maternal microbiome on offspring brain development and behaviour^18^. Taken together, based on the phenotype of F1-S and F2 males, we concluded that the hypoactivity trait is also transmitted by iterative somatic programming. Consistent with somatic transmission, the hypoactivity phenotype was not transmitted to F2-G males (Fig. 1h).

An additional variable receptor-associated non-genetic trait is reduced immobility/increased escape-directed behaviour in the forced swim test^15^. Although traditionally interpreted as an ‘antidepressant'-like behaviour, it more likely reflects increased reactivity to a stressful environment^15^. Alternatively, it may reflect a preference for an active, as opposed to passive, coping strategy to a strong stressor^19^. Although weak and below significance in F1 and KO males, the escape response was robust in F2 males (Fig. 1i,j). F1 and F2 females also exhibited a phenotype, but in the opposite direction, indicating a sex difference (Fig. 1k). We have previously reported that the increased stress-reactivity phenotype was absent in F1-S males^15^, suggesting that the trait is not somatically transmitted. Here we show that F2-G males have increased stress-reactivity (Fig. 1l), indicating that the transmission of this trait is gametic. A lack of the increased stress-responsive phenotype in F1 males (born to H mothers) but the presence in both F2 and F2-G males (born to WT mothers) supports the idea that the H gestational/postpartum environment moderates this gametically programmed phenotype. The increased stress-reactivity phenotype, seen in F2-G males was not apparent in the next generation (Supplementary Fig. 3), indicating that gametic programming of this phenotype is not strictly transgenerational.

**Figure 2:**
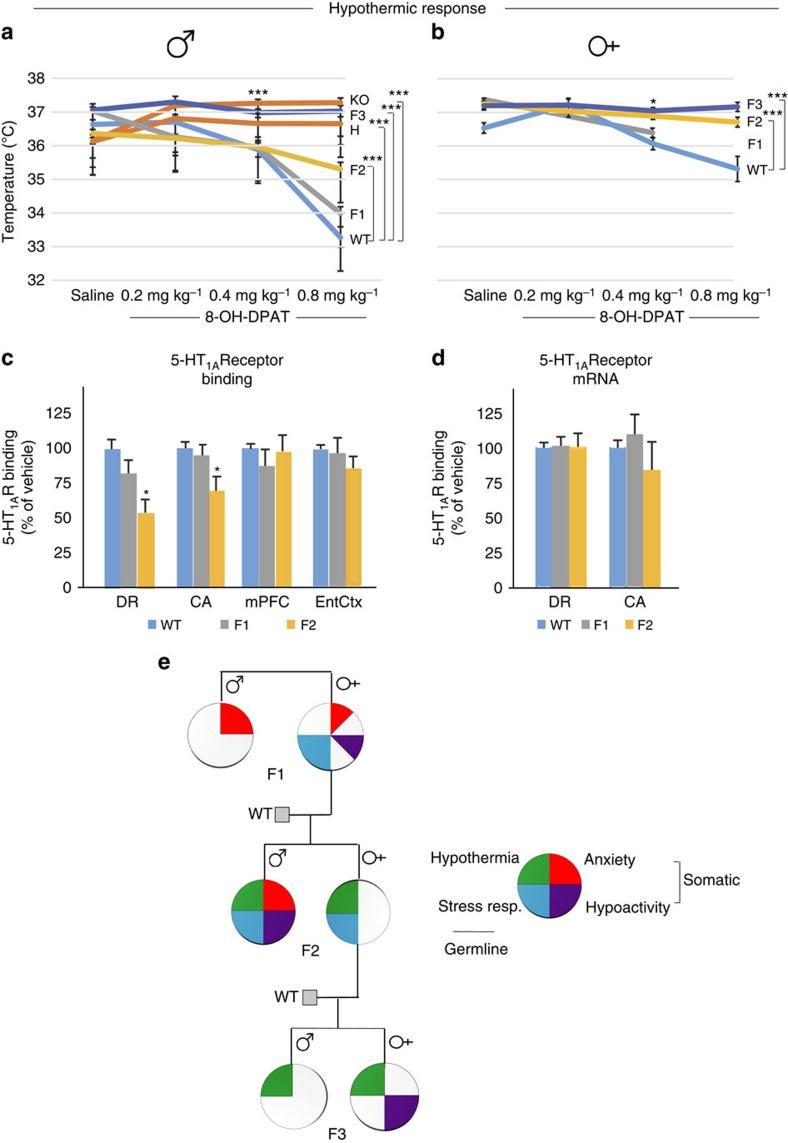
Non-genetic transmission of trait associated with the lack of drug-induced hypothermia and summary of transmission of all traits. (**a**) F2, F3, H and KO males exhibit blunted response to the 5-HT_1A_R agonist 8-OH-DPAT (repeated-measures analysis of variance (ANOVA): group, F_5,86_=23.9, *P*<10^−6^; dose, F_3,258_=67.9, *P*<10^−6^; and group × dose, F_15,258_=29.7, *P*<10^−6^; LSD *post hoc* ***P<0.005, relative to WT at the same dose; *N*=WT 19, F1 13, F2 23, F3 12, H 15, KO 16). (**b**) F2 and F3 females also exhibit blunted hypothermia response to drug (repeated-measures ANOVA: group, F_2,24_=13.7, *P*=0.0001; dose, F_3,72_=17.1, *P*<10^−6^; and group × dose, F_6,72_=7.6, *P*=0.000002; LSD *post hoc* *P<0.05, ***P<0.005, relative to WT at the same dose, *N*=WT 6, F1 8, F2 10, F3 11). (**c**) F2 males have reduced [^3^H]-8-OH-DPAT binding (ANOVA: DR, F_2,15_=6.37, *P*=0.01; CA, F_2,15_=4.46, *P*=0.030; LSD *post hoc* *P<0.05, *N*=6 for all groups), but (**d**) not receptor mRNA expression. CA, CA subfields of the hippocampus; DR, dorsal raphe; EntCtx, entorhinal cortex; mPFC, medial prefrontal cortex. (**e**) Variable penetrance, gender specificity and different modes of transmission of traits generate a highly variable phenotype in the pedigree. Each quarter circle represents significant phenotype, whereas trend is represented by one-eighth of a circle.

Surprisingly, we also found that, while WT mice responded to the 5-HT_1A_R agonist 8-OH-DPAT with hypothermia, F2 and F3 males and females, similar to H or KO mice, had a partially/completely blunted drug response (Fig. 2a,b). The hypothermic response of 8-OH-DPAT is mediated by 5-HT_1A_ presynaptic autoreceptors in the raphe nucleus^20^. Embryo transfer itself induced blunted hypothermic response (presumably because of the surgical/transplantation procedure(s) and the resultant effect on the mother and/or embryo), preventing us from determining the exact transmission mechanism of this particular trait (Supplementary Fig. 4). Nevertheless, these data suggest that thermoregulation can be perturbed by the environment, revealed by a receptor agonist. To directly test if the blunted drug response in F2 males was due to reduced receptor availability in the raphe, we measured receptor binding by using [^3^H]-8-OH-DPAT. Indeed, F2 males had significantly reduced receptor binding in the dorsal raphe nucleus (Fig. 2c and Supplementary Fig. 5). F1 males, consistent with their drug-induced hypothermia response, had normal receptor binding. In F2 males, binding was also reduced in the hippocampus, expressing 5-HT_1A_ postsynaptic receptors, indicating that the non-genetic effect is not restricted to the presynaptic pool. However, levels of postsynaptic receptors in two cortical areas were unchanged in F2 animals. As *in situ* hybridization and RNA-Seq showed no receptor mRNA changes in F2 dorsal raphe and hippocampus (Fig. 2d), the absence of drug-induced hypothermia/5-HT_1A_R-binding is not based on a direct transcriptional mechanism, but presumably on impaired translation and/or receptor trafficking/coupling, which can be based on either a 5-HT_1A_R-specific or a broader mechanism.

Schematic representation of the transmission of the four traits in Fig. 2e underscores the variable- and sex-specific penetrance of the associated behaviours, resulting in behavioural pleiotropy in the pedigree. The anxiety-like behavioural trait is characterized by sex-specific and limited intergenerational transmission, as it was present in males up to the F2 generation, but absent in females, except a trend for increased innate fear in F1 females. In contrast, the other traits seem to be partially penetrant but multigenerational because they were weak or not expressed in F1, expressed robustly in F2 (especially in males), and were occasionally transmitted to the F3 generation. Importantly, the traits were transmitted and/or expressed independently from each other across the generations, even the somatically programmed anxiety-like and hypoactivity traits, indicating their segregated transmission.

### Transmission of the somatic anxiety-like behavioural trait

**Figure 3:**
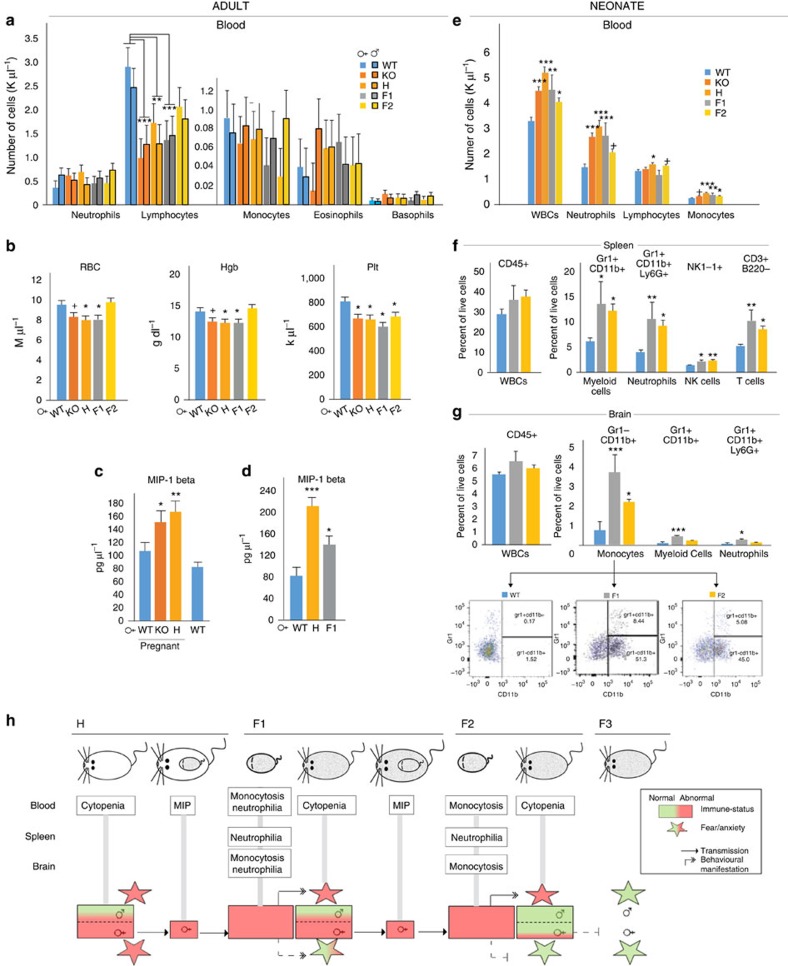
Immune system dysregulation in H and F1 females is associated with immune activation in their offspring and the transmission of somatic anxiety trait. (**a**) Lymphocytopenia in KO, H, F1, but not F2, adult females and males (analysis of variance (ANOVA). Lymphocyte Group Effect, F_4,30_=4.482, *P*=0.006; Lymphocyte Sex, F_1,30_=0.338, *P*=0.566; LSD *post hoc* *P<0.05, **P<0.01, ***P<0.005, *N*=8 per group/4 each sex). (**b**) Anaemia with low haemoglobin (Hgb) levels in KO, H, F1, but not F2, females and thrombocytopenia in KO, H, F1 and F2 adult females (ANOVA. red blood cells (RBCs): F_4,15_=4.199, *P*=0.018; Hgb: F_4,15_=3.592, *P*=0.030; platelets (Plt): F_4,15_=4.568, *P*=0.013; LSD *post hoc* *P<0.05, **P<0.01, ^+^*P*<0.01 (trend only), *N*=4 per group). (**c**,**d**) Increased plasma MIP-1β levels in gestational day 10 KO and H dams measured by multiplex bead-based immunoassay (**c**, ANOVA F_3,16_=7.662, *P*=0.002; LSD *post hoc* *P<0.05, **P<0.01, *N*=5 per group) and in adult KO, H and F1 females by ELISA (**d**, ANOVA F_2,6_=16.745, *P*=0.004; LSD *post hoc* *P<0.05, ***P<0.005, *N*=3 per group). (**e**,**f**). Blood and spleen of F1 and F2 P3 neonates have increased number of neutrophils and monocytes (blood, ANOVA: F_4,72_=9.99, *P*=0.000002; LSD *post hoc* ***P<0.01, trend ^+^*P*<0.10, *N*=15, 22, 15, 8 and 14; spleen, ANOVA: F_2,12_=6.53, *P*=0.012; LSD *post hoc* **P<0.01 and *P<0.05, *N*=6, 3 and 6). (**g**) Brains of F1 and F2 P3 neonates have transmigrated monocytes (ANOVA: F_2,12_= 10.07, *P*=0.0027; LSD *post hoc* ***P<0.005 and *P<0.05, *N*=6, 3, 6). Lower panels show the gating strategy. (**h**) Summary of maternal and neonatal immune abnormalities across generations. Adult female cytopenia and increased MIP-1β are associated with the transmission of the anxiety-like trait, whereas neonatal immune abnormalities in the blood, the spleen and the brain are associated with the sex-specific development of anxiety in males.

To gain insight into the mechanism of somatic programming, we focused on the anxiety-like phenotype because it was robustly transmitted to F1 and F2 males. We hypothesized that transmission of this particular trait from mother to offspring is through a brain-immune-brain pathway for the following reasons: (i) a deficit in brain 5-HT_1A_Rs is associated with depression and stress disorders^13^, conditions that can lead to a proinflammatory state in both mother and offspring^21^ and (ii) immune activation in the offspring can result in abnormal emotional behaviours^22^. First, we asked if H females exhibit signs of an immune system abnormality that is transmitted to F1 females (because their F2 male offspring have anxiety-like behaviour), but not to F2 females (because their F3 offspring do not display anxiety-like phenotype). Complete blood count (CBC) showed a reduced number of lymphocytes in KO, H, F1, but not F2 adult females, and similarly in adult males (Fig. 3a). In addition to the lymphocytopenia, KO, H, F1, but not F2, females exhibited a lower than normal number of red blood cells (accompanied by low haemoglobin level), suggesting anaemia (Fig. 3b). Finally, all groups (KO, H, F1 and F2) showed thrombocytopenia (Fig. 3b). In contrast to females, the number of red blood cells and platelets was normal in adult H, F1 and F2 males (not shown, Fig. 3h). Although elucidating the mechanism that initiates anaemia, lymphocytopenia and thrombocytopenia in 5HT_1A_R receptor-deficient H females will require additional research, it is known that cytopenias are often secondary to immune activation. For example, pancytopenia has been reported following severe viral infection such as HIV, likely caused by immune activation as a result of the indirect systemic effects of virus replication^23^. However, KO, H and F1 females were kept in a specific pathogen free (SPF) facility and showed no signs of infection. Other pathological conditions with anaemia, lymphocytopenia/leukopenia and thrombocytopenia are autoimmune diseases, particularly systemic lupus erythematosus (SLE)^24^. SLE is characterized by waxing and waning flares of inflammation and immune activation, resulting in the production of autoantibodies that target various organs and cells, including red blood cells, lymphocytes/leukocytes and/or platelets^25^. Given the more profound cytopenia in H and F1 female mice (anaemia, lymphocytopenia and thrombocytopenia), as compared with H and F1 males (lymphocytopenia only), it is interesting to note that SLE, and autoimmune diseases in general, are known to be more prevalent in females than in males^26^. Overall, these studies suggest a complex immune/haematological condition in females that is closely associated with the maternal transmission of the anxiety-like phenotype (in H and F1 females), a notion strengthened by the simultaneous cessation of anaemia and lymphocytopenia and the transmission of the anxiety-like behaviour (in F2 females; Fig. 3h).

Cytopenias are often secondary to immune activation, and immune activation during gestation, via proinflammatory cytokines, can programme behavioural alterations in the offspring^10^,^22^. Such mechanism would be consistent with the prenatal origin of offspring anxiety-like phenotype shown by crossfostering and embryo transfer studies in our previous report^15^. Therefore, we next tested the level of 35 cytokines/bioactive compounds in the plasma of gestational day 10 KO and H mothers by microsphere-based immuno-multiplexing technology (Myriad RBM, Mouse CytokineMAP A, B, C). One cytokine, macrophage inflammatory protein 1β (MIP-1β), but not MIP-1α, IL-1β or IL-6 among others, was increased in both pregnant KO and H mothers as compared with WT pregnant females (pregnancy had no effect on MIP-1β levels in WT; Fig. 3c). The increase in plasma MIP-1β was replicated by ELISA (R&D Systems Mouse CCL4/MIP-1 beta Quantikine) in H and was also found in F1 females (not pregnant; Fig. 3d). MIPs are produced by macrophages, dendritic cells and lymphocytes and have chemotactic and proinflammatory effects via the G-protein coupled receptor (GPCR) CCR5 (ref. 27). Blocking CCR5 by maraviroc^28^ in H mothers through pregnancy (300 μg ml^−1^ in drinking water) resulted in no change in male offspring anxiety-like behaviour (distance travelled in elevated plus maze; *t*-test: *T*=−1.195, *P*=0.246, *N*=10 drug, 11 vehicle), suggesting that the elevated MIP-1β level in H/F1 mothers may not contribute or more likely is not solely sufficient to programming anxiety. Nonetheless, the increased MIP-1β levels are indicative of immune activation/proinflammatory state in H and F1 mothers (Fig. 3h).

Because gestational immune activation produces offspring immune dysregulation and later life behavioural abnormalities^10^,^22^, we next assessed the immune system of 3-day-old neonates, born to KO, H and F1 mothers. Their KO, H, F1 and F2 offspring all had elevated levels of white blood cells (WBCs) in peripheral blood (Fig. 3e). Within WBCs, the number of neutrophils was increased in H and F1, with a trend in F2 pups, whereas the number of monocytes was increased in all H, F1 and F2 groups, suggesting a proinflammatory state (Fig. 3e). Neonates had normal red blood and platelet counts (not shown). Flow analysis of neonatal spleens showed similar increases in myeloid and neutrophil cells, together with an elevated number of NK and T cells, in both F1 and F2 neonates (Fig. 3f), but the spleens of adult H, F1 and F2 mice were normal (not shown). In addition, leukocytosis in F1 and F2 pups was associated with the transmigration of a significant number of Gr1^−^CD11b^+^ monocytes to brains suggesting their activated state (Fig. 3g). F1 neonatal brains also contained elevated numbers of Gr1^+^CD11b^+^ myeloid cells and Gr1^+^CD11b^+^Ly6G^+^ neutrophils. We found no immune cell transmigration in adult brain (not shown) suggesting that the abnormal migration to the brain is limited to the developmental period. This finding may provide a plausible mechanism to explain the anxiety-like phenotype of F1 and F2 males because the presence of activated monocytes and their development to macrophages in brain parenchyma have been shown to cause anxiety-like behaviour in mice^29^ (Fig. 3h). Minimal anxiety or lack thereof in F1 and F2 females could be due to sex-dependent differences in transmigration and/or its impact on brain development, a notion consistent with epidemiological and preclinical research indicating the unique vulnerability of males to prenatal adversity^30^.

Taken together, adult female/mother immune abnormalities strongly correlated with the transmission of the anxiety-like trait to their F1 and F2 male offspring, whereas immune activation and brain-transmigration of activated monocytes in neonates aligned with the later development of male-specific anxiety behaviour.

### Transcriptome and metabolome changes across generations

Next, we tested if somatic programming is associated with persistent gene expression changes in the brains of F1 and F2 males. Lesion, pharmacological and optogenetic studies link anxiety-like behaviour in the elevated plus maze to dentate gyrus granule cells, particularly those in the ventral hippocampus^31^,^32^. Consistent with this association, we reported delayed granule cell maturation, specifically in the ventral hippocampus, in F1 offspring^15^. In turn, delayed hippocampal development may result in persistent transcriptional abnormalities that could contribute to the F1 adult anxiety-like phenotype.

**Figure 4:**
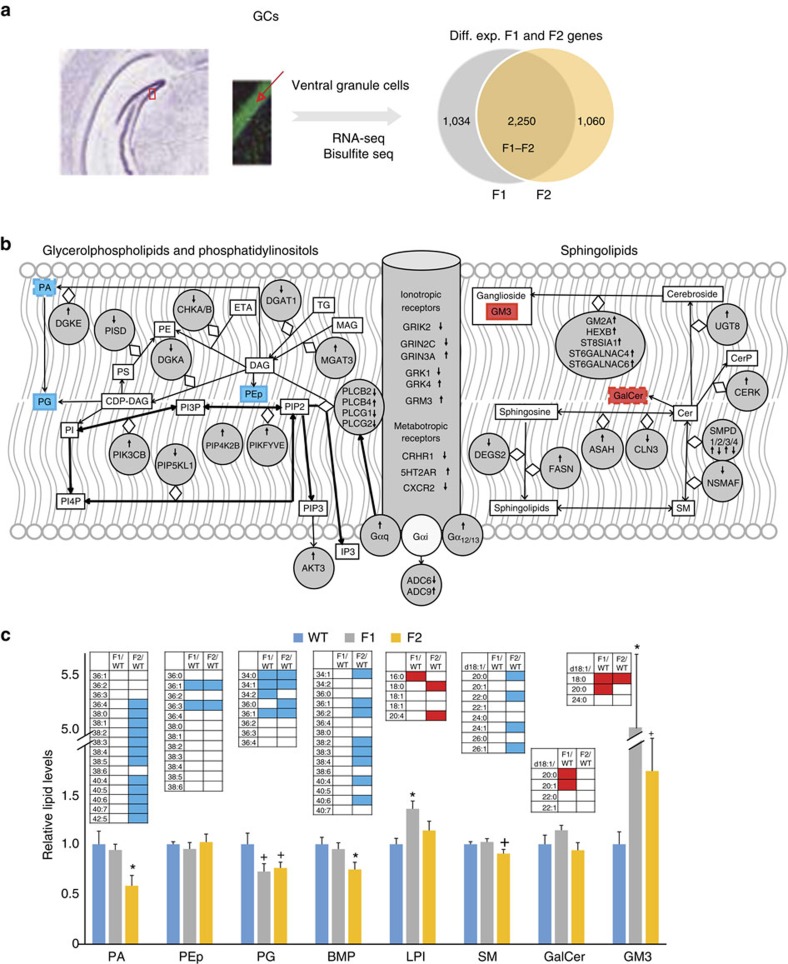
The impact of the receptor-deficient maternal environment on the F1/F2 neuronal transcriptome and metabolome. (**a**) A sagittal section of the ventral hippocampus highlighting the location of ventral granule cells, the source of mRNAs and metabolites. Granule cells can be isolated as a homogenous population because of their clustering in the granule cell layer (cells are highlighted by their green NeuN-positive nuclei). Only a few cells positive for the glia marker glial fibrillary acidic protein (GFAP) present within the granule cell layer (blue cytoplasmic staining). Most of the differentially expressed F1 and F2 genes overlap. (**b**) Ingenuity functional analysis of differentially expressed overlapping F1–F2 genes identified enrichment in functions related to sphingolipid metabolism (right), receptor signalling (centre) and glycerophospholipids (left), indicated by solid grey circles, as a result of the maternal effect. Gene/protein nomenclature, see Supplementary Table 2. Lipid changes shown in **c** are also highlighted (blue box represents reduced and red increased levels in both F1/F2 granule cells, whereas red/blue boxes with staggered outline represent changes in either F1 or F2. Cer, ceramide; DAG, diacylglycerol; ETA, ethanolamine; GalCer, galactosylceramide, GM3, monosialodihexosylganglioside; MAG, monoacylglycerol; PA, phosphatidic acid; PE, phosphatidylethanolamine, PEp, plasmalogen phosphatidylethanolamine; PG, phosphatidylglycerol; PI, phosphatidylinositol; PS, phosphatidylserine; SM, sphingomyelin. (**c**) Glycerophospholipid and sphingolipid composition of F1 and F2 ventral granule cells, relative to WT cells. BMP, bis(monoacylglycero)phosphate; LPI, lysophosphatidylinositol. *N*=6 per group. Columns represent total levels of lipid subclasses. Results are presented as mean and bars as s.e.m, *P≤0.05; and trend ^+^*P*≤0.1 by analysis of variance with Bonferroni *post hoc* test (*α*: 0.0166). Blue and red boxes in insets represent significantly reduced and increased levels in specific lipid species within lipid subclasses; see also Supplementary Table 4 for the extent of changes.

The high density and clustering of granule cell bodies in the granule cell layer of the dentate gyrus and their separation from most other cell types (Fig. 4a) allowed us to microdissect them from cryosections and then assess gene expression, as a function of prior maternal environment. RNA-Seq identified ∼3,000 differentially expressed genes in both F1 and F2 adult males (false discovery rate (FDR) *q*<0.01), with a significant (2/3) overlap, consistent with the shared anxiety-like phenotype of these mice (Fig. 4a). Surprisingly, differentially expressed genes in both F1 and F2 (same direction of change) were primarily enriched in membrane lipid-related functions, including *sphingolipid*, glycerophospholipid (that is, *phosphatidylethanolamine)* and *diacylglycerol metabolism* (Ingenuity Functional Analysis, *P*=0.000037, 0.00019 and 0.0045, respectively; Supplementary Table 1). Although anxiety is typically viewed as a dysfunction in neurotransmission, membrane lipids and their metabolizing enzymes are known to regulate membrane receptor function and intracellular signalling and have been implicated in psychiatric diseases, including anxiety^33^,^34^.

Differentially expressed genes encoded key enzymes in sphingolipid metabolism (a total of 11; Fig. 4b and Supplementary Table 2). Regulation was complex, with both up- and downregulation. Interestingly, we also found seven genes, all upregulated, that are involved in the metabolism of the glycosphingolipid GM3 (Supplementary Table 2), suggesting a possible co-regulation of these genes. Additional differentially expressed genes were associated with glycerophospholipid metabolism. Moreover, ingenuity analysis identified the canonical *Superpathway of Inositol Phosphate Compounds* (*P*=0.00014, within the glycerophosholipid network in Fig. 4b). Phosphoinositol signalling is triggered by receptor activation, and we also found a number of differentially expressed genes encoding membrane receptors, including GPCRs and their downstream signalling molecules (Supplementary Table 1 and Fig. 4b). In sum, the transcriptome data suggest a broad dysfunction in lipid signalling in F1 and F2 ventral granule cells.

Next, we performed untargeted profiling of 3,156 metabolites in granule cells from individual WT and F1 mice that identified 21 differentially expressed metabolites (*P*≤0.05), of which 11 were structurally defined, and confidently distinguished F1 from WT granule cells (Supplementary Fig. 6 and Supplementary Table 3). Remarkably, 9 out of 11 of the structurally defined molecules were recognized as membrane/bioactive lipids, comprised of 5 glycerophospholipids (phosphatidic acid 43:2, phosphatidylethanolamine 38:5, phosphatidylcholine 38:6, diacylglycerol 40:8 and 40:7), 3 lactosylceramides (differing in carbon chain length and degree of unsaturation) and 1 lipid metabolite (3-phosphoglyceroinositol), all diminished by 10–90% in F1 as compared with WT granule cells.

A more comprehensive targeted lipidomic study assessed the levels of a large number of lipid species in both F1 and F2 granule cells. Phosphatidic acid levels (measured in the range of 30-42 in total fatty acid chain length) again were reduced, but reached statistical significance only in F2 neurons (Fig. 4c and Supplementary Table 4), which is in line with the generally more robust behavioural phenotype of the F2 offspring (Fig. 2e). Phosphatidic acid is known to play major roles in regulating membrane trafficking and cellular signalling through interaction with effector proteins or by direct effects on lipid bilayers^35^. Levels of specific plasmalogen phosphatidylethanolamine (36:1 and 36:3) and phosphatidylglycerol (34:0, 34:1, 36:1) species were reduced in both F1 and F2 granule cells. Levels of some bis(monoacylglycerol)phosphate species were also generally reduced, but reached statistical significance only in F2. In contrast, different lysophosphatidylinositol species were increased in both F1 and F2 neurons. Although untargeted metabolomics suggested reductions in the levels of specific molecular species of phosphatidylcholine and diacylglycerol as well, these changes were not verified by targeted lipidomics (however, the diacylglycerol species identified in untargeted metabolomics were not analysed in the targeted lipidomics experiment). In sum, levels of major glycerophospholipids were reduced in F1 and F2 neurons, in some cases only in F2 neurons (Fig. 4c). Regarding sphingolipids, sphingomyelin levels were reduced, but only in F2 neurons, whereas galactoceramide levels were increased in F1 GCs. However, levels of another glycosphingolipid, GM3, were increased in both F1 and F2 neurons and represented the largest changes in the F1/F2 lipidome (Fig. 4c). GM3 and other sphingolipids are enriched in raft-like microdomains, specialized membrane domains where transmembrane signalling occurs through receptors and associated signalling components^36^. Taken together, the deficit in major glycerophospholipids and the surplus in some glycosphingolipids in F1/F2 granule cells highlight an imbalance between two major classes of membrane lipids. These data, in combination with the transcriptional findings, strongly implicate abnormal neuronal signalling through altered lipid metabolites and/or lipid–receptor interactions in F1 and F2 granule cells, possibly underlying the iterative anxiety-like phenotype.

### DNA methylation changes co-segregate with the anxiety-like phenotype

**Figure 5:**
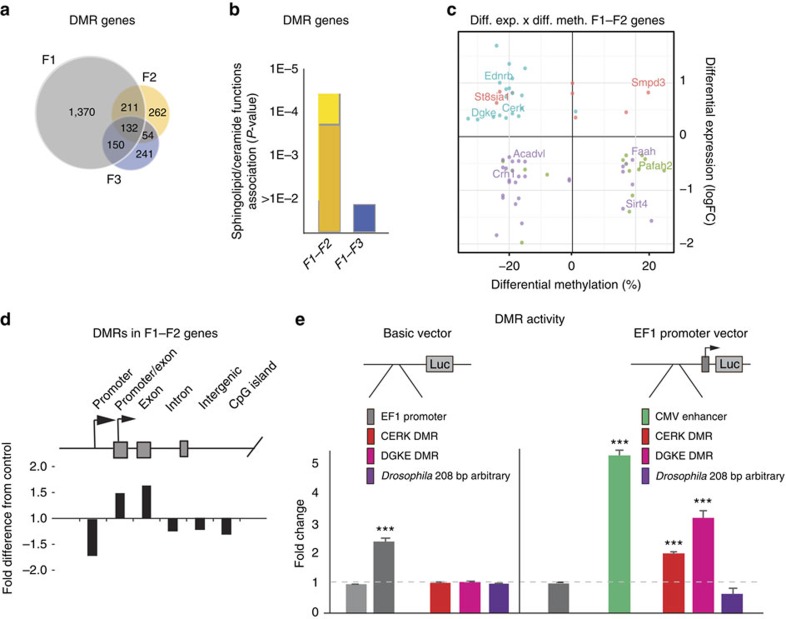
Impact of the receptor-deficient maternal environment on the F1/F2 neuronal methylome. (**a**) Overlap of genes with differentially methylated regions (DMRs) in F1, F2 and F3 ventral granule cells. (**b**) Overrepresentation of DMR genes in F1 and F2, but not in all F1, F2 and F3 granule cells, in the process of *sphingolipid/ceramide*-related processes (see list of genes in Supplementary Table 5). The short and long columns indicate the lowest and highest significance levels for these processes. (**c**) Similar changes in DNA methylation and gene expression in F1 and F2 neurons. F1 and F2 overlapping differentially methylated and expressed genes are distributed to four quadrants, according to their expression (up/downregulated) and average methylation at differentially methylated sites within DMRs (hyper/hypomethylated; +/−). Colour of genes is rendered according to their differential expression/differential methylation values in F1 (blue, up/hypo quadrant; purple: down/hypo; green, down/hyper and red, up/hyper), whereas their actual positions reflect values in F2. Most genes in a quadrant are of a similar colour, indicating similar expression and methylation changes in F1 and F2 neurons. (**d**) F1/F2 overlapping DMRs are overrepresented at exonic regions. (**e**) Selected DMRs from *Cerk* and *Dgke* (Fig. 4b) have no promoter, but significant enhancer-like, effects on the EF1 promoter, similar to that of the cytomegalovirus (CMV) enhancer, in transfected 293 cells (analysis of variance: F_4.49_=163.16, *P*<10^−4^; LSD *post hoc* ***P<0.005 for both CERK and DGKE, in addition to the positive control CMV). An intergenic *Drosophila* sequence has neither promoter nor enhancer activity.

We have previously reported DNA methylation changes in adult F1 ventral granule cells^37^. Here we profiled F1, F2 and F3 ventral granule cells from adult males and followed the segregation of methylation signatures with the anxiety-like phenotype across generations. Methylation at ∼1.5 million CpGs and ∼7 million non-CpGs was measured genome wide by bisulfite sequencing at relatively CpG-rich and gene-rich regions, including ∼2/3 of the promoters, ∼¾ of the CpG islands and a substantial fraction of exons^38^,^39^. Differentially methylated sites (≥15%, *q*<0.01) tended to be clustered (≥4), forming differentially methylated regions (DMRs, ∼200–300 bp). DMRs were present in all F1, F2 and F3 neurons (Fig. 5a). The change in methylation occurred mostly at CpG dinucleotides (>95%), either hypomethylation or hypermethylation (55% and 45%), and was unidirectional in all differentially methylated sites within 87% of the DMRs. Thus, the methylation landscape in F1–F3 neurons was characterized by mostly uniformly hypomethylated or hypermethylated DMRs distributed across the genome.

Most DMRs (>80%) mapped to intragenic sequences and could therefore be unambiguously assigned to specific genes. We hypothesized that DMRs and their genes are associated with anxiety if present in both F1 and F2 granule cells, whereas the set of DMR genes present in all F1, F2 and F3 neurons may lack key differentially methylated genes required for the development of the anxiety-like behaviour (F3 males do not exhibit anxiety). Approximately half of the 659 F2 DMRs was also present in F1 neurons, of which the majority was absent in F3 neurons, demonstrating the existence of a substantial pool of genes that meets the criteria for association (Fig. 5a). As Fig. 5b shows, functional analysis of DMRs present in both F1 and F2 neurons identified *sphingolipid/ceramide*-related processes (Supplementary Table 5) and included genes previously identified by the transcriptome analysis. In contrast, these functions were not enriched in DMR genes present across all the F1+F2+F3 neurons. These data indicate the co-segregation of differentially methylated membrane lipid genes with the anxiety-like phenotype and further strengthen the association between these genes and the phenotype, first suggested by the transcriptome and lipidomics studies.

Besides membrane lipid-related functions, F1–F2 shared DMR genes were also enriched in neurotransmission and synaptic functions (Supplementary Table 5). Furthermore, the F1–F2 DMR gene data set also identified *anxiety* (*P*=0.00002), the very trait that was transmitted to the F1 and then to the F2 generation, as the top associated behaviour (Supplementary Table 5). The broader functional categories identified by differential DNA methylation, relative to differential expression (limited to lipids, Supplementary Table 1), could be due to persistent hypo/hypermethylation from early life at genes that regulate synaptic development but which are no longer required in mature neurons, or simply to subtle or isoform-specific changes that were not detected in our transcriptome studies.

Next, we intersected the DNA methylation and gene expression data and again identified *sphingolipid*- and *glycerophospholipid*-related functions (Supplementary Table 6). There was no direct correlation between methylation and expression changes (Fig. 5c); not an unexpected finding given that both positive and negative correlations have been reported with intragenic methylation^40^,^41^,^42^. However, these data showed that the direction of expression changes was identical, and the overall methylation changes were largely the same, in F1 and F2 neurons, indicating that programming of the transcriptome and methylome, similar to that of the anxiety-like behaviour, is iterative.

### Anxiety-associated DMRs have enhancer-like activity

As most DMRs were intragenic, assignment of DMRs to specific gene features may shed light on their function. As Fig. 5d shows, F1–F2 shared DMRs were enriched in exons, including those that map to alternative promoters, but excluded from proximal promoters, introns and intergenic areas. DMRs from *Cerk* and *Dgke*, two representative DMRs (∼200 bp), from differentially expressed and methylated genes associated with lipid signalling (Fig. 4b), were selected for further studies. They reside within H3K4me3/H3K27ac/H3K9ac and H3K27ac/H3K4me1 peaks, respectively, in both neuronal (cerebellar neurons) and non-neuronal cells (ENCODE^43^). These chromatin signatures are typically associated with promoter and enhancer sequences. In a luciferase reporter assay, these DMRs exhibited no promoter activity, while enhancing the activity of the EF1 promoter in 293 cells (Fig. 5e). An arbitrary 208 bp intergenic *Drosophila* sequence had neither promoter nor enhancer activity. These data suggest that at least some of the DMRs represent regulatory elements associated with enhancers and that their enhancer-like activity is not limited to neurons and could contribute to gene regulation in multiple tissues.

### DNA methylation at gametically programmed genes

**Figure 6:**
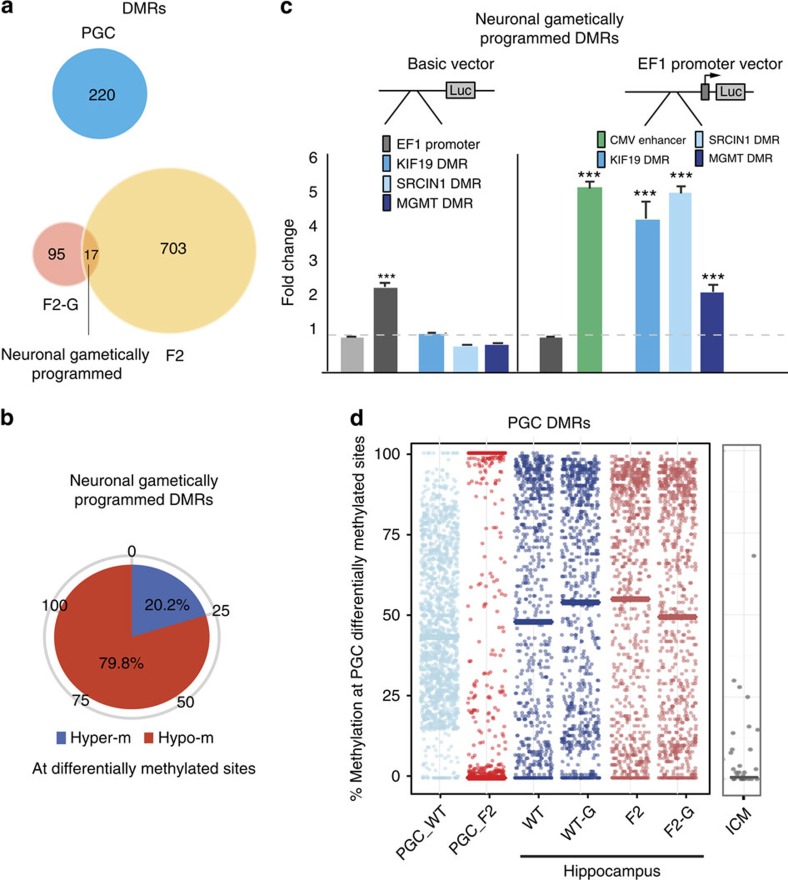
Characteristics of gametically programmed DMRs. (**a**) PGC DMRs are unrelated to neuronal gametically programmed DMRs, which are defined as overlapping DMRs in F2 and F2-G neurons. (**b**) Methylation changes at neuronal gametically programmed sites are predominantly hypomethylation. (**c**) Neuronal gametically programmed DMRs have no promoter, but significant enhancer, activity (analysis of variance: F_5,57_=170.82, *P*<10^−4^; LSD *post hoc* ***P<0.005 for KIF19, SRCIN1 and MGMT). (**d**) Distribution of methylation levels at individual CpG sites, differentially methylated in F2 PGCs, in PGCs, hippocampal granule cells and inner cell mass (ICM).

Altered stress-reactivity of the F2 offspring in the forced swim test was gametically transmitted through the maternal line, which prompted us to identify gametically programmed neuronal-DMRs (Fig. 1i,l). We reasoned that gametically programmed neuronal DMRs should be present in the brain of two independent sets of animals with H grandmaternal ancestry, F2 and F2-G, both exhibiting increased stress-reactivity (Fig. 1i,l). F2 mice, derived by normal breeding, should carry both somatic and gametically programmed DMRs, whereas F2-G animals, obtained through embryo transfer, should harbour only gametically programmed DMRs and perhaps DMRs that are produced by the embryo transfer procedure^44^. By overlapping these two DMR sets, one can identify common DMRs that are linked to gametic programming. This approach identified 17 overlapping DMRs (false discovery rate (FDR) *q*<0.05, overlap Fisher exact test *P*=0.0065, odds ratio 4.11), mostly hypomethylated (80%), representing 21 genes (Fig. 6a,b). These data also suggest that the much larger group of F2 DMRs represents mostly non-gametic (that is, somatic) DMRs (see also the large set of F1-F2 iterative DMRs, Fig. 5a). Although the low number of gametic DMRs prevented us to analyse their association with genomic features, three representatives of neuronal gametic DMRs, located at distal promoter/exon sequences, all enhanced the activity of the EF1 promoter in luciferase assays (Fig. 6c), indicating that, similar to somatic DMRs, they represent regulatory elements, presumably enhancers.

Unlike somatic, neuronal gametically programmed DMR genes were not enriched in membrane lipid signalling/neurotransmission-related functions. Rather, 9 out of 21 of these genes mapped directly to synaptic morphology-related functions and/or neuropsychiatric risk genes. Specifically, *Disc1* and *Srcin1* are involved in spine maintenance and morphogenesis, respectively (Ingenuity *P*=0.00085 and 0.0034); *Itga6* in neurite morphogenesis (*P*=0.00085); *Gng7* and *Soc5* in transmembrane signalling (*P*=0.0084 and *P*=0.025), *Kif19* in microtubule dynamics (*P*=0.00338); whereas *Disc1*, *Pias4*, *Rsrc1* and *Frem* have been implicated in autism, schizophrenia and neurological disorders. Intriguingly, *Disc1* was also associated with *adaptive behaviour* and *immobility in the forced swim test*^45^ (*P*=0.00069 and *P*=0.0014), the very trait identified as gametically programmed. However, escape directed behaviour in the forced swim test has been linked to multiple brain regions, including the prefrontal cortex, raphe and the hippocampus; thus, it will be important to test if the hippocampal gametically programmed DMRs are also present in other relevant brain regions.

Next, we asked if the neuronal gametically programmed DMRs can be traced back to female primordial germ cells (PGCs). We isolated E18.5 F2 and WT PGCs from pregnant H and WT mothers by FACS^46^ and identified 220 DMRs (Fig. 6a). However, none of these F2 PCG-DMRs matched the brain DMRs. Moreover, F2 PGC-DMRs did not propagate to somatic/neuronal cells because methylation at these genomic locations was similar in hippocampal neurons, independent of whether the offspring were the granddaughter of WT or H mothers (Fig. 6d, WT-G, F2-G). Finally, methylation at F2 PGC-DMRs was almost completely lost at the blastocyst stage, consistent with extensive reprogramming in the early embryo (Fig. 6d, right column). Taken together, these data indicate that the H grandmaternal environment results in hypomethylation at a small number of DNA regions in PGCs. However, these methylation differences are not permanent because of the loss of methylation at affected CpG sites during early embryonic reprogramming, followed by normal developmental remethylation.

As F2 PGC-DMRs may alter embryonic development, that in turn produce the neuronal DMRs^47^, we mapped them to genes and functions. DMR-associated genes were enriched in few functions, most prominently in *imprinting* (*P*=0.00034) and *fertility* (*P*=0.00042), encompassing some of the same genes (Supplementary Table 7).These DMR genes regulate proliferation, growth and differentiation and imprinted genes are known to be essential in early embryonic growth and development^48^. Although none of the DMRs mapped to imprinted regions, they nevertheless could alter the expression level of the active alleles.

## Discussion

Here we address a central problem in the epidemiology and diagnosis of complex disorders; particularly, how their symptoms are propagated across generations, even in the absence of plausible genetic factors^49^. We also explore why they may present differently in individuals, even those in the same family tree. For example, family members can have various combinations of symptoms from anxiety, depression, bipolar disease and schizophrenia, complicating diagnosis and treatment.

Although the behavioural manifestations associated with a genetic risk factor are transmitted together via that genetic factor, here we show that the individual non-genetic traits of a complex psychiatric disease-like phenotype are propagated across multiple generations independently, by parallel non-genetic mechanisms. The ‘anxiety' and ‘hypoactivity' traits were transmitted by a somatic mechanism, whereas the ‘increased stress-reactivity' trait was transmitted by a gametic, mechanism. In humans, these traits transcend diagnostic categories and are found in comorbid generalized anxiety and depression disorders, as well as other psychiatric conditions. As the individual traits/pathways each have their own generation-dependent penetrance and gender specificity, the resulting cumulative phenotype is pleiotropic. Pleiotropy, which refers to the ability of a single gene or factor to produce multiple phenotypic outcomes^50^, is a commonly occurring phenomenon in psychiatric disorders^51^, making diagnosis and treatment difficult. In the context of genetic diseases, it is typically assumed that this phenomenon arises from individual differences in vulnerability to the various effects of the causative gene. However, the work presented here reveals that pleiotropy can be produced by the variable distribution and segregated transmission of behavioural traits.

Although some of the maternal traits propagated to the F2 and even to the F3 generation (that is, hypoactivity), contrary to our expectation, this was not based on a true transgenerational mechanism, but was rather due the reiteration of single generational somatic transmission, referred here to as iterative somatic transmission. The iterative propagation of the male-specific anxiety-like behaviour is most compatible with a model in which proinflammatory state is propagated from H to F1 females and in which the proinflammatory state is acquired by F1 males from their H mothers, and then by F2 males from their F1 mothers. We propose that increased levels of gestational MIP-1β in H and F1 mothers, together with additional proinflammatory cytokines and bioactive proteins, are required to produce immune system activation in their newborn offspring, which in turn promotes the development of the anxiety-like phenotype in males. In particular, increase in the number of monocytes and their transmigration to the brain parenchyma in F1 and F2 males could be central to the development of anxiety. Monocytes develop to macrophages in the brain and activate resident microglia, as reported in various animal models of psychiatric disorders^22^,^29^,^52^. Because microglia participate in programmed cell death, survival, axon remodelling, pruning and synaptogenesis in various brain regions, including the hippocampus^53^, it is reasonable to hypothesize that the increased number of peripheral monocytes and their transmigration to the brain contributes, via microglia activation, to the development of anxiety-like phenotype in the F1 and F2 male offspring. The hypoactivity trait also followed a somatic programming scheme, but its manifestation was highly variable across generations, which contributed to the pleiotropy.

The somatically programmed behaviours, in particular, the anxiety-like behaviour that can be linked to the ventral hippocampus, were accompanied by DNA methylation changes that converged on the functional networks of lipid signalling and synaptic/neurotransmission in hippocampal granule cells. Corresponding transcriptional and lipidomic changes further advocate for the role of the lipid signalling network in somatic programming. However, complexity and high redundancy in the regulation of lipid metabolic pathways^54^ complicate linking the specific transcriptional changes directly to the lipid alterations. Nonetheless, studies implicate membrane lipids and their metabolites in psychiatric diseases, including anxiety^33^,^34^.

Embryo transfer experiments showed that the increased stress-reactivity trait was propagated to the F2 generation by gametic transmission in our model. This indicates that gametically programmed information survives reprogramming in the early embryo and persists into adulthood. However, it was erased in the F3 germline, meaning the transmission is not strictly transgenerational. Although we identified gametically programmed DMRs in both F2 PGCs and adult hippocampal neurons, their dissimilarity indicates that they are either unrelated or, as suggested in other models^55^, the primary PGC epigenetic signatures may initiate a cascade of developmental changes in the early zygote and/or later development, leading to secondary epigenetic signatures in adult tissues. Indeed, PGC DMRs were associated with imprinted genes that are known to be essential in early growth and development^48^. Alternatively, epigenetic, other than DNA methylation, changes could be formed in PGCs at neuronal DMR regions that later, during neuronal development, acquire the adult neuronal DNA methylation signatures. Whether derived from PGC DMRs indirectly or from other ‘primed' regions, neuronal gametically programmed DMRs map to genes whose dysfunctions can be linked to the altered stress-reactive phenotype.

In summary, our data introduce segregated non-genetic transmission of traits as a mechanism that may explain the non-Mendelian propagation and pleiotropy of behavioural/psychiatric phenotypes across generations. A transmission-pathway-based concept complements the current gene-centred view of inheritance in psychiatric disease, as well as facilitate the development of animal models with better construct validity^56^ and the identification of new drug targets.

## Methods

### Animals

Animal experiments were carried out in accordance with the Weill Cornell Medical College Institutional Animal Care and Use Committee guidelines. All mice were group housed up to five per cage with 12-h light/dark cycle with lights on at 0600 hours. Food and water were available *ad libitum*. 5-HT_1A_R KO mice were originally generated on the 129SvEv background^14^ and backcrossed to the Swiss–Webster (SW, Taconic Biosciences) background >10 times. We used the outbred SW background (a strain often used in behavioural experiments), to avoid the possible contribution of homozygous genetic variants in inbred strains to behavioural phenotypes^57^. Of note, the H line was backcrossed every five to ten generations to WT mice obtained from large colonies kept at Taconic Biosciences, and the H and WT lines were re-established, to avoid genetic drift. The F1 generation consisted of littermate *5-HT*_*1A*_*R*^+/+^ (F1-WT; F1), *5-HT*_*1A*_*R*^+/−^ (H, heterozygote) and *5-HT*_*1A*_*R*^−/−^ (KO) mice (generated as previously described^14^,^15^), all exposed to the receptor-deficient H maternal environment. F1 females were crossed with WT males to generate F2 offspring (all WT). F2 females were crossed with WT males to generate F3 offspring. A separate WT lineage, bred with the F1–F3 lines in parallel, provided control WT offspring exposed to normal, WT gestational environment. Since only 25% of pups derived from H × H crosses are WT (that is, F1), and because genders were separately analysed, on average, only one F1 male and one F1 female were obtained from a H mother. As a comparable number of mothers were used for all groups, the number of offspring was similar to that of the litter across all groups. Each behavioural experiment was repeated with three cohorts, typically representing a total of >9 litters/offspring.

### Behavioural procedures

All tests were conducted using offspring aged 8–12 weeks. During all behavioural tests, the investigators who performed the tests were blind to the genotype and treatment of the animals. Moreover, all behavioural tests are fully automatized with no manual data collection. Behavioural tests were conducted during the light on phase, between 1000 hours and 1600 hours. Mice were first tested in open field for overall activity, followed 24 h later by elevated plus maze and 3 days later by the forced swim test. The drug-induced hypothermia tests were conducted a minimum of 1 week after the forced swim test. All tests were conducted between 0900 hours and 1800 hours (that is, during the light cycle). Animals with a behavioural measure of greater or smaller than mean±2 s.d. were excluded. The elevated plus maze^15^ was performed using a cross maze with 12 × 2 inch arms at dim light (50 lux). Animals were introduced to the middle portion of the maze facing an open arm and allowed to freely explore for 10 min. Time spent and distance travelled in the open and closed arms were measured by a video-tracking system (Noldus Information Technology). We found ‘distance travelled' in the open arm as the most reproducible and valid measure of open arm activity because it is not confounded by the repeated entry into and immediate freezing in the open arm of some animals that, by inflating open arm time and entry, can lead to the underestimation of anxiety levels. The open-field test^15^ used a 15 × 21 inch black box, divided into 12 even-sized (4 × 3 inch) rectangles. The time spent and distance travelled in the two rectangles at the centre of the field at 150 lux were recorded by the video-tracking system to evaluate anxiety, and data were presented as a percentage of total distance travelled. In the forced swim test^15^, mice were placed into a clear, water-filled cylinder (diameter, 20.3 cm; depth, 10 cm), essentially as described by Porsolt *et al*.^58^ In this test, immobility of the mice is scored by an observer in 2 min bins for a total of 6 min. On test days, animals were transported to the dimly illuminated behavioural laboratory and left undisturbed for at least 1 h before testing.

### Hypothermic response

Hypothermic response^59^ was assessed at different dosages of 8-OH-DPAT over the course of 4 days because the response does not show habituation. Animals were individually housed 1–2 h before the start of the experiment. On day 1, animals were injected (i.p.) with 100 μl saline. After 30 min, rectal temperature was measured. On days 2–4, mice were administered 0.8, 0.4 and 0.2 mg kg^−1^ of 8-OH-DPAT, respectively, before taking temperature measurements.

### Embryo transfer

Two types of embryo transfers were performed^15^. SW WT embryos (E0.5–E2.5) were implanted into pseudopregnant 5-HT_1A_R^+/−^ females (7- to 9-week-old) to generate F1-S (somatically exposed) offspring. To generate F2-G (gametically exposed) offspring, embryos (E0.5–E2.5), derived from female PCGs/oocytes exposed to the 5-HT_1A_R^+/−^ grandmaternal environment (within the WT F1 female fetus) were implanted into pseudopregnant SW WT females (7- to 9-week-old).

### 5-HT_1A_R receptor binding and *5-HT*_*1A*_ *R* expression

Tissue preparation, *in situ* hybridization and receptor autoradiography procedures were performed as previously described^59^. Briefly, mice were euthanized by pentobarbital overdose and brains rapidly removed, frozen on dry ice and stored at −80 °C. Coronal tissue sections (14 μm) were cut using a microtome-cryostat (HM500-OM, Microm), thaw-mounted onto 3-aminopropyltriethoxysilane (Sigma-Aldrich)-coated slides and kept at −20 °C until use. For 5-HT_1A_R mRNA, antisense oligoprobe was complementary to bases 1,780–1,827 (GenBank accession NM_008308). Oligonucleotide was labelled (2 pmol) at the 3'-end with [^33^P]-dATP (>2,500 Ci mmol^−1^; DuPont-NEN) using terminal deoxynucleotidyltransferase (TdT, Calbiochem). The autoradiographic binding assays for 5-HT_1A_R was performed using [^3^H]-8-OH-DPAT (233 Ci mmol^−1^). Autoradiograms were analysed and relative optical densities (ROD) were obtained using a computer-assisted image analyser (MCID). The system was calibrated with ^3^H-microscales standards to obtain fmol/mg protein equivalents from ROD data. The slide background and nonspecific densities were subtracted. ROD were evaluated in two or three adjacent sections by duplicate of each mouse and averaged to obtain individual values.

### Complete blood count

All neonatal CBCs were performed on pups between 3 and 4 days old. Blood was collected from the trunk via decapitation. Total red blood cells, WBCs, neutrophils, lymphocytes, monocytes, eosinophils and basophils were quantified by both automated (IDEXX ProCyte Dx Hematology Analyzer) and manual counts.

### Flow cytometry

The presence of immune subsets in peripheral blood, spleens and brains was assessed in adult and 3- to 4-day-old pups by flow cytometry. All animals were anaesthetized and perfused with saline in order to flush out circulating blood cells from the organs. Spleen cells were collected by homogenization and filtering through a 40-μm cell strainer into FACS buffer (PBS with 0.5% BSA). Brains were harvested in FACS buffer followed by three sequential digestion steps in RPMI+25 mM HEPES+Collagenase D (1 mg ml^−1^)+DNAase 1 (0.2 mg ml^−1^) at 37 °C for 5 min each. Spleen and brain single-cell suspensions were counted using a Z2 Coulter Counter (Beckman Coulter) and subsequently stained with a 10-colour panel with the following antibodies: Ly6G-FITC, Gr1-PE, NK1.1-PerCP-Cy5.5, CD11c-PE-Cy7, CD11b-APC (all from BD Biosciences) and CD45-APC-Cy7, CD3-BV570, B220-BV650, CD4-BV711 and CD8-BV780 (all from BioLegend). Samples were acquired within an hour after staining in an LSR-II flow cytometer (BD Biosciences) and analysed on FlowJo 9.7.6 software.

### Isolation of PGCs

PGCs were isolated from E18.5 F1 and WT fetal ovaries based on side and forward scatter differences between germ cells and somatic cells in cell suspension, essentially as described in ref. 46. This method provides higher than 90% purity of isolated PGCs, which was verified in our experiments by stationing sorted cells for VASA, a gemline-specific marker. Following sorting of PGCs from individual embryos, the genotype of the embryo was determined, and only F2 PGCs from WT F1 fetuses were used for DNA isolation. Because of the relative low number of germ cells per embryo, 45 F1 and 31 WT female fetuses were used.

### Enhanced reduced representation bisulfite sequencing

Brains from adult male WT, F1, F2, F3, WT-S, F1-S and F2-G offspring (8- to 12-week-old) from at least three different litters were quick frozen on dry ice and sectioned (200 μM) in cryostat. The ventral dentate gyrus was microdissected from the sections. DNA was isolated using the DNeasy Blood & Tissue Kit (Qiagen). Single end 50 bp ERRBS sequencing was performed as described^39^, using Illumina HiSeq2000 and 2,500 machines according to the manufacturer's instructions. In a previous report, we demonstrated the correspondence of ERRBS methylation data, based on chemical bisulfite Illumina sequencing, with methylation data obtained by the methylation-sensitive enzyme-based HELP assay, as well as by bisulfite-Massarray sequencing^37^. An in-house ERRBS pipeline (R MethylKit) was used for methylation calling and alignment to the mm9 reference genome^60^. To test reproducibility, ventral granule cells from six mice were individually bisulfite sequenced, which revealed a high concordance of methylation status between the replicates.

Differentially methylated sites tended to cluster in groups of ≥4 in areas of few hundred basepair long. These clusters were not enriched in CpG islands as they typically had lower CpG content and resided distal to transcriptional start sites (Fig. 5d). As we aimed to identify DMRs that reflect the actual location of differentially methylated sites and include changes in both directions rather than an arbitrary window size with a difference in average methylation, we chose to define DMRs as regions containing a minimum of four differentially methylated sites, either at CpG or non-CpG (CpH) dinucleotides, where the corrected *P*-value was ≥0.01 (0.05 in the gametically programmed data) and the difference in methylation between two samples was ≥15%. We selected the distance between two differentially methylated sites to be no greater than 1 kb, but the actual distance was only 40–60 bps. The reproducibility of this approach was demonstrated by the high concordance of F1 DMRs in duplicate samples. Genomic and CpG island annotations were based on Ensmbl data downloaded from the University of California Santa Cruz (UCSC) genome browser. Promoters were defined as regions ±2 kb from the transcription start site (TSS); exons and introns were defined by reference; upstream regions were defined as extending 50 kb from promoter regions; and downstream regions were 50 kb from the transcription end site (TES). The percentage of total differentially methylated sites in a defined genomic feature was divided by the percentage expected to overlap each genomic feature by chance, based on the percentage of genomic space occupied by that feature, to determine the fold change from expected values. Additional methylation data sets were downloaded from the UCSC genome browser DNA methylation track hub^61^,^62^,^63^. Data have been deposited: GSE68713.

### RNA-Seq

WT, F1 and F2, adult offspring (8-to 12-week-old) from at least three different litters were perfused with 30% RNAlater (Ambion) in saline. Brains were frozen on dry ice and sectioned (200 μM). The ventral dentate gyrus was microdissected from the sections. Total RNA was isolated using the RNeasy Mini Kit (Qiagen). Single end 50 bp RNA sequencing was performed on Illumina HiSeq2000 and 2,500 machines and aligned to the mm9 reference genome using TopHat software version 2.0.11 (ref. 64). Default parameters were used with the addition of ‘--no-novel-juncs' to align exclusively to known genes and isoforms. Genes were counted using HT-seq program^65^ with the parameter ‘intersection-strict'. Values for gene expression were calculated using EdgeR^66^ package in R using tagwise dispersion and default parameters. Differentially expressed genes were determined using Benjamini–Hochberg corrected *P*=0.05 threshold. Data have been deposited: GSE68713.

### Transfection and luciferase reporter assay

Exonic DMRs and a control *Drosophila* sequence were amplified by PCR (Droso208Forward Sequence: 5′-CATAGTACTAGGATCCACGCCTAAAGCAACTCCAC-3′, Droso208Reverse: 5′-GTTACATGTTGGATCCAGGGATGGGCGTTGGAGA-3′; CERK F: 5′-CATAGTACTAGGATCCGAACTGGGTAATTTTGTTTGT-3′, CERK R: 5′-GTTACATGTTGGATCCCCTCCTCTGTGTTCTCAG-3′; DGKE F: 5′-CATAGTACTAGGATCCCACGCCAGCAATGCTTGC-3′, DGKE R: 5′-GTTA CATGTTGGATCCGTGCTCCGTGCTGTTGCC-3′; Kif19 F: 5′-CATAGTACTAGGATCGAGGGCCAGAGCCTCAGA-3′, Kif19 R: 5′-GTTACATGTTGGATCATGCCAAACCTGAACCTGG-3′; SRCIN1 F: 5′-CATAGTACTAGGATCCTGGTCTCTGTGTCTTTGGC-3′, SRCIN1 R: GTTACATGTTGGATCCCACCCCCGCACAGCCCCT-3′; MGMT F: 5′-CATAGTACTAGGATCCGGGCCCGCAGCCTGCTAA-3′, MGMT R: 5′-GTTACATGTTGGATCCTTGAGCCAGGTCCCAGTC-3′. Plasmids were constructed using the In-Fusion Cloning Kit (*Clontech* Laboratories). HEK293 cells (American Tissue Type Collection) were cultured in culture media containing 88% DMEM+10 mM HEPES, 1% penicillin/streptomycin, 10% FBS and 1% L-glutamine in a 5% CO_2_ 37 °C incubator. Plasmids were transfected into HEK293 cell cultures using Lipofectamine Transfection Reagent (Life Technologies) in triplicates. Briefly, 0.2E6 cells were seeded in 12-well plates in 1 ml of culture media. Twenty-four hours later, 1 μg of plasmid DNA was diluted in 85 μl Opti-MEM media (Life Technologies) in tubes. After 5 min incubation, 6 μl Lipofectamine Transfection Reagent was added, followed by 20 min incubation. Eighty-five microlitres of this solution was added to each well of HEK293 cells. The following day, media were aspirated and replaced with 400 μl of culture media. Forty-eight hours after transfection, 20 μl aliquots of media were sampled into 96-well plates. One hundred microlitres of Quanti-Luc luciferase substrate (InvivoGen) was added to each well, and plates were read immediately for luciferase activity.

### Metabolomics

*Brain metabolite extraction*. Hippocampal ventral granule cell bodies/nuclei were isolated by microdissection from adult male WT and F1 brain sections and washed twice with ice-cold PBS, followed by metabolite extraction using −70 °C 80% methanol in water (LC-MS grade methanol, Fisher Scientific, Grand Island, NY). The tissue–methanol mixture was subjected to bead-beating for 45 s using a *Tissuelyser* cell disrupter (Qiagen). Extracts were centrifuged for 5 min at 5,000 r.p.m. to pellet insoluble material and supernatants were transferred to clean tubes. The extraction procedure was repeated two additional times and all three supernatants were pooled, dried in a *Vacufuge* (Eppendorf) and stored at −80 °C until analysis. The methanol-insoluble protein pellet was solublized in 0.2 M NaOH at 95 °C for 20 min and quantified using the Bio-Rad DC assay (Bio-Rad). On the day of metabolite analysis, dried cell extracts were reconstituted in 70% acetonitrile with 0.2% ammonium hydroxide at a relative protein concentration of 3 μg ml^−1^ and 3 μl of the reconstituted extract was injected for LC/MS-based untargeted metabolite profiling.

*LC/MS metabolomics platform for untargeted metabolite profiling*. Brain extracts were analysed by LC/MS as described previously^67^,^68^ using a platform comprised of an Agilent Model 1200 liquid chromatography system coupled to an Agilent 6230 time-of-flight MS analyser. Chromatography of metabolites was performed using aqueous normal phase gradient separation, on a *Diamond Hydride* column (Microsolv). Mobile phases consisted of: (A) 50% isopropanol, containing 0.025% acetic acid and (B) 90% acetonitrile containing 5 mM ammonium acetate. To eliminate the interference of metal ions on the chromatographic peak integrity and electrospray ionization, EDTA was added to the mobile phase at a final concentration of 6 μM. The following gradient was applied: 0–1.0 min, 99% B; 1.0–15.0 min, to 20% B; 15.0–29.0, 0% B; 29.1–37 min, 99% B. Raw data were analysed using MassHunter Profinder 6.0 and MassProfiler Professional 13.0 software package (MPP; Agilent Technologies). Unpaired *t*-tests (*P*<0.05) were used to determine significant differences between groups.

*Differentially expressed metabolite identification*. To ascertain the identities of differentially expressed metabolites (*P*<0.05) between F1 cross versus WT vGC samples, molecular features were searched against an in-house *METLIN* Personal Metabolite Database (Agilent Technologies), annotated with accurate monoisotopic neutral masses (<5 p.p.m.) and chromatographic retention times. A molecular formula generator algorithm in MPP was used to generate and score empirical molecular formulae based on a weighted consideration of monoisotopic mass accuracy, isotope abundance ratios and spacing between isotope peaks. A tentative compound ID was assigned when *METLIN* and molecular formula generator scores concurred for a given candidate molecule. Tentatively assigned molecules were verified based on a match of LC retention times and/or MS/MS fragmentation spectra to that of pure molecule standards.

### High-performance liquid chromatography–mass spectrometry

Tissue lipid extracts from isolated ventral granule cells were prepared using a modified Bligh/Dyer procedure, spiked with appropriate internal standards, and analysed using a 6490 Triple Quadrupole LC/MS system (Agilent Technologies). Glycerophospholipids and sphingolipids were separated with normal-phase HPLC as before^69^,^70^ with a few changes. An Agilent Zorbax Rx-Sil column (inner diameter 2.1 × 100 mm^2^) was used under the following conditions: mobile phase A (chloroform/methanol/1 M ammonium hydroxide, 89.9:10:0.1, v/v) and mobile phase B (chloroform/methanol/water/ ammonium hydroxide, 55:39.9:5:0.1, v/v); 95% A for 2 min, linear gradient to 30% A over 18 min and held for 3 min, and linear gradient to 95% A over 2 min and held for 6 min. Sterols and glycerolipids were separated with reverse-phase HPLC using an isocratic mobile phase as before^69^ except with an Agilent Zorbax Eclipse XDB-C18 column (4.6 × 100 mm^2^). Individual lipid species were measured by multiple reaction monitoring transitions and lipid concentration was calculated by referencing to appropriate internal standards: D5-cholesterol, cholesterol ester 17:0, 1,2-diphytanoyl-sn-glycero-3-phosphocholine 16:0 diether diacylglycerol, D5-TAG 16:0/18:0/16:0, phosphatidic acid 14:0/14:0, phosphatidylcholine 14:0/14:0, phosphatidylethanolamine 14:0/14:0, phosphatidylglycerol 15:0/15:0, phosphatidylserine 14:0/14:0, lysophosphatidylcholine 17:0, lysophosphatidylethanolamine 14:0, lysophosphatidylinositol 13:0, bis(monoacylglycero)phosphate 14:0/14:0, sphingomyelin d18:1/12:0, dihydrosphingomyelin d18:0/12:0, ceramide d18:1/17:0, galactosylceramide d18:1/12:0, lactosylceramide d18:1/12:0 and sulfatide d18:1/17:0 (Avanti Polar Lipids). Phosphatidylinositol 16:0/16:0 was purchased separately (Echelon Biosciences). Some lipid classes did not have commercially available internal standards and hence these lipids were referenced to standards that are closely eluted in the liquid chromatography–mass spectroscopy method: Ether-linked species were normalized to corresponding acyl-linked standards: GM3 to phosphatidylinositol 16:0/16:0. Lipid concentration was normalized by molar concentration across all species for each sample, and the final data are presented as mean mol%.

### Data analysis

Data are shown as mean±s.e.m. Outlier data were excluded based on±2 s.d. from the mean. One-way or repeated-measures analyses of variance or *t*-tests were used to compare tests. Least significant difference (LSD) or Bonferroni *post hoc* analyses were used to assess statistical significance. Sample size was based on prior data and by using power calculation All graphs and statistical analysis were performed using R (http://ww.r-project.org), Bioconductor (http://www.bioconductor.org) and ggplot2 (http://www.ggplot2.org) for visualization, unless stated otherwise.

## Additional information

**How to cite this article**: Mitchell, E. *et al*. Behavioural traits propagate across generations via segregated iterative-somatic and gametic epigenetic mechanisms. *Nat. Commun.* 7:11492 doi: 10.1038/ncomms11492 (2016).

## Supplementary Material

Supplementary Information Supplementary Figures 1-6, Supplementary Tables 1-7
