# Supplementary material for: Behavioural traits propagate across generations via segregated iterative-somatic and gametic epigenetic mechanisms
Source: Nat Commun. 2016 May 13;7:11492. doi: 10.1038/ncomms11492 (PMC4869176; doi:10.1038/ncomms11492)
Supplement: Supplementary Information — Supplementary Figures 1-6, Supplementary Tables 1-7 [file ncomms11492-s1.pdf]

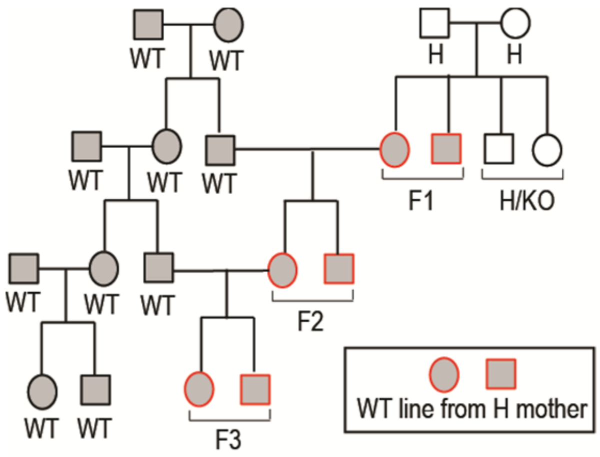

Supplementary Figure. 1. Breeding strategy of F1-F3 WT, derived from H mothers and matched WT animals, derived from WT mothers. Shaded boxes indicate genetically WT individuals.

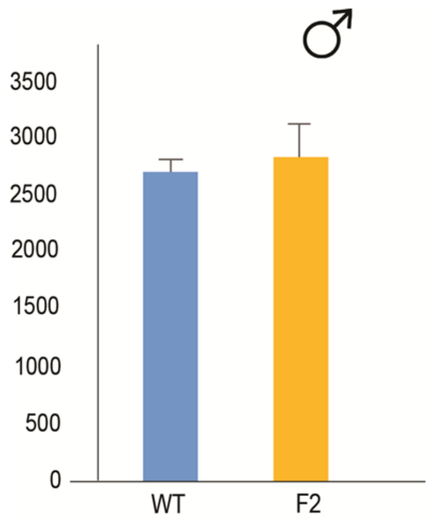

Supplementary Figure. 2. Total locomotor activity of F2 males are not different from that of WT males, *t*-test, p=0.645. N=15 and 11.

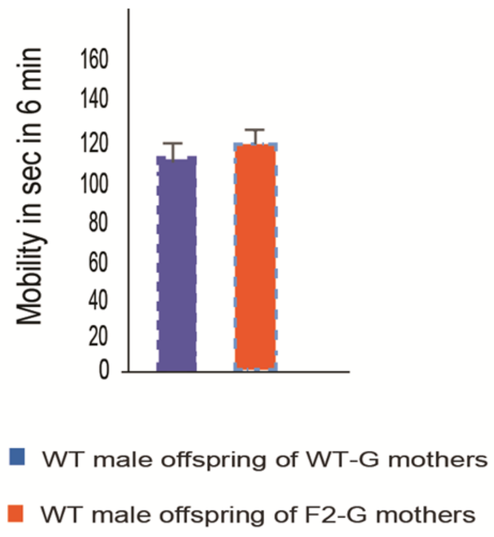

Supplementary Figure. 3. The increased stress reactivity phenotype seen in F2-G males is not transmitted to the next generation. N=30 and 15.

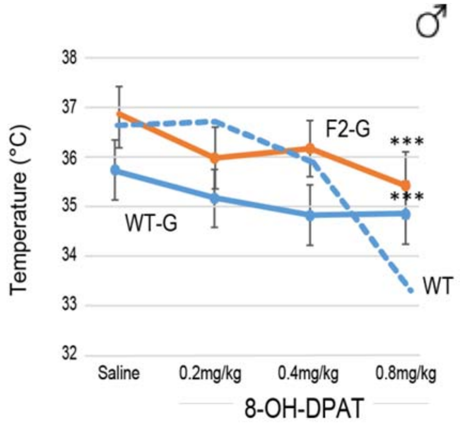

Supplementary Figure. 4. Blunted hypothermic response to the 5-HT_{1A}R agonist 8-OH-DPAT following embryo transfer in both F2-G mice and their WT-G controls (repeated measures ANOVA: group, $F_{2,129}$=10.8, P=0.0002; dose, $F_{3,129}$=24.4, $P<10^{-6}$; and group x dose, $F_{6,129}$=8.4, $P<10^{-6}$; LSD *post hoc* ***p<0.005 for both groups, relative to WT at the same dose. N=18, 12, and 10 animals per group).

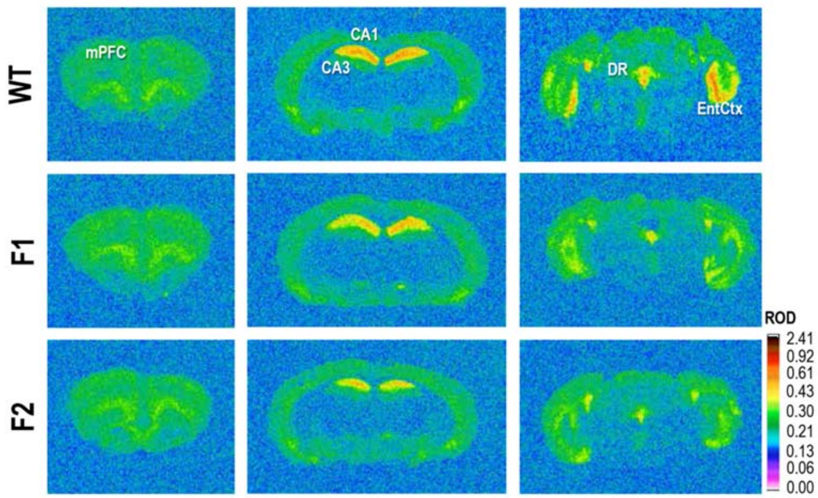

Supplementary Figure. 5. Representative coronal brain sections showing reduced 5-$HT_{1A}R$ expression in hippocampus and dorsal raphe nuclei assed by [^{3}H]-8-OH-DPAT binding.

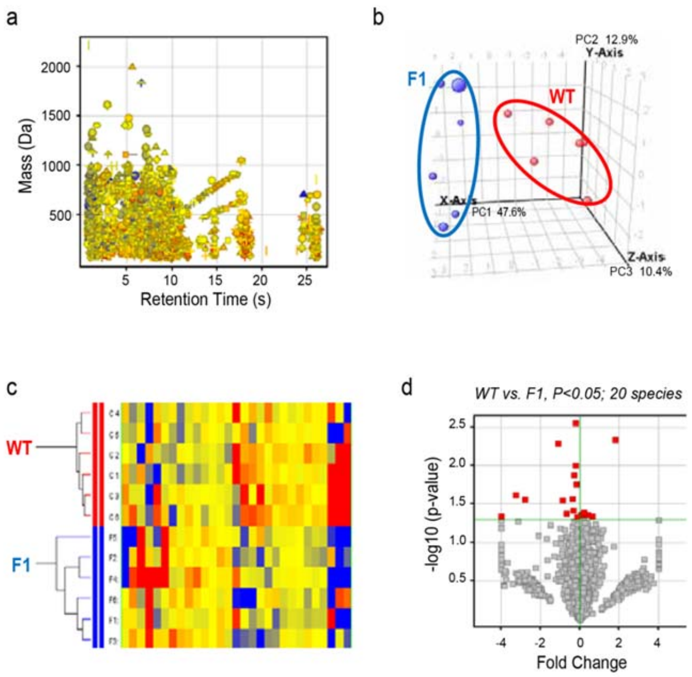

Supplementary Figure. 6. Untargeted metabolite profiling identifies differentially-expressed metabolites in GCs from F1 vs. WT mice (n = 6 mice/group). **a**. A total of 3,156 distinct molecular features were aligned and quantified by untargeted molecular feature extraction in all samples from at least one group, as either positive ions (1,726) or negative ions (1,503). **b**. Principal component analysis (PCA) shows within group clustering and between group separation of GC extracts from WT and F1 brains, considering the relative abundances of 21 differentially-expressed features from among the 3,156 species in panel a ($P < 0.05$). Each point corresponds to an individual mouse brain extract. **c**. Unsupervised hierarchical clustering analysis (HCA) according to Euclidean distance metric and Ward's linkage rule with samples color-coded by phenotype, displays expression patterns and clustering of the differentially-expressed metabolites ($p<0.1$). Feature intensity depicted as a heat map, ranging from red to blue, where red represents an expression level greater than the mean value and blue represents expression levels below the mean value. **d**. Volcano analysis of all 3,156 detected brain features, where between group differences in metabolite expression are plotted at the -log of fold-change vs. log of the p-value. Notably, of the 21 molecules that fulfill the criteria of differential expression with a p-value ≤ 0.05 (indicated in red), 9 were identified as lipids with diminished abundance in F1 vs. WT brain samples while 11 were not structurally confirmed (see Extended Data Table 4).

Supplementary Tables

Supplementary Table 1. Functions enriched in overlapping F1 and F2 differentially expressed genes

Functional Annotation	p-Value	Molecules	# Molecules
hydrolysis of lipid	3.75E-05	ABHD4,ACAA1,AGT,ARFRP1,ARRB2,CHRM2,CXCR2,ENPP6,FAAH,GBA2,GM2A,GNA12,GNAQ,GPT,GRK4,HEXB,HTR2A,IKBKB,LDL, LPL,MGLL,NAAA,NSMAF,PAFAH1B1,PAFAH2,PLA2G3,PLCB4,PLCG1,PLCG2,PNPLA6,PPT1,PRKAA2,RGS4,SMPD1,SMPD2,UNC5B	36
neuronal ceroid lipofuscinosis	6.10E-05	CLCN7,CLN3,CLN5,CTSD,PPT1,SMPD1,TPP1	7
synthesis of glycosphingolipid	6.65E-05	ABCA8,ABCB1,BCL2L1,CD82,CERK,CLN3,DEGS2,FASN,LARGE,NSMAF,PRKCD,PRKDC,SCD,SLC27A1,SMPD1,SMPD2,SMPD3,SMP D4,ST6GALNAC6,ST8SIA1,UGT8	21
metabolism of sphingomyelin	7.76E-05	CLN3,SMPD1,SMPD2,SMPD3,SMPD4	5
synthesis of glycolipid	7.89E-05	ABCA8,ABCB1,BCL2L1,CD82,CERK,CLN3,DEGS2,FASN,GPAA1,HEXB,LARGE,NSMAF,PIGL,PIGO,PRKCD,PRKDC,SCD,SLC27A1,SM PD1,SMPD2,SMPD3,SMPD4,ST6GALNAC6,ST8SIA1,UGT8	25
cleavage of lipid	8.03E-05	ABHD4,ACAA1,AGT,ARFRP1,ARRB2,CHRM2,CXCR2,ENPP6,FAAH,GBA2,GM2A,GNA12,GNAQ,GPT,GRK4,HEXB,HTR2A,IKBKB,LDLR ,LPL,MGLL,NAAA,NSMAF,PAFAH1B1,PAFAH2,PLA2G3,PLCB4,PLCG1,PLCG2,PNPLA6,PPT1,PRKAA2,PTGS1,RGS4,SMPD1,SMPD2, UNC5B	37
metabolism of sphingolipid	1.31E-04	ABCA8,ABCB1,BCL2L1,CD82,CERK,CLN3,DEGS2,FASN,GBA2,GM2A,HEXB,LARGE,NSMAF,PPT1,PRKCD,PRKDC,SCD,SLC27A1,SM PD1,SMPD2,SMPD3,SMPD4,ST6GALNAC6,ST8SIA1,UGT8	25
synthesis of phosphatidylethanolamine	1.93E-04	CHKA,CHKB,PCYT2,PISD,SLC27A1	5
sialylation of ganglioside	6.02E-04	ST6GALNAC4,ST6GALNAC6,ST8SIA1	3
concentration of lipid	8.64E-04	ABCA7,ABCB1,ABCC8,ACP6,ADIPOR2,AGT,AKT3,ALDH1A1,AMH,APAF1,ARNT,BCAT2,BCL2L1,CASP9,CERK,CHKA,CHRNA7,CLN3, CLU,CRHR1,CRTC2,CTSD,CXCL12,CYP27B1,DAGLB,DGAT1,DGAT2,DGKE,DHCR24,DIO2,DLG2,DLK1,DNM2,EDN3,EPB41,FA2H,FA AH,FADS2,FAM57B,FASN,FGF7,GAD2,GBA2,GNAQ,GPR116,GPX1,HEXB,HMGA1,HMGCR,HSD3B7,IL18,IL18BP,INHA,INPP5B,INSIG 1,IRS2,LBP,LDLR,LPGAT1,LPL,MAG,MGAT3,MGLL,MRAS,NAA40,NEIL1,NKX2- 1,NPC1L1,NSMAF,NTRK2,PCYT2,PDGFRA,PEX5L,PIKFYVE,PIP5KL1,PLA2G3,PLD2,PLIN5,PLP1,PLSCR3,PNPLA6,PNPLA8,PPP1R3C ,PRKAA2,PRKCD,PROKR2,PTGS1,PTPN11,QKI,RGS4,RORA,RPS6KB2,SAFB,SAT1,SCD,Scd2,SCG5,SCNN1A,SIRT6,SLC1A2,SLC27 A1,SLC6A11,SLCO2A1,SMPD1,SMPD2,SMPD3,SOAT2,ST8SIA1,TAZ,TGFA,TGFBR2,TRIB1,TYK2,UCP2,UGT8,UHMK1,WNK4	117
hydrolysis of phospholipid	1.23E-03	AGT,ARRB2,CHRM2,CXCR2,ENPP6,GNA12,GNAQ,GPT,GRK4,HTR2A,LPL,NSMAF,PAFAH1B1,PAFAH2,PLA2G3,PLCB4,PLCG1,PLCG 2,PNPLA6,RGS4,SMPD1,SMPD2,UNC5B	23
synthesis of ceramide	2.73E-03	BCL2L1,CERK,CLN3,DEGS2,FASN,NSMAF,PRKCD,PRKDC,SCD,SMPD1,SMPD2,SMPD3,SMPD4	13
quantity of polyunsaturated fatty acids	3.34E-03	AGT,CERK,DAGLB,DGKE,DNM2,FAAH,FADS2,GNAQ,GPX1,IL18,IL18BP,LDLR,LPL,PNPLA8,PTGS1,SCD,SLCO2A1,TAZ,TGFA,TGFB R2	20
metabolism of phospholipid	3.35E-03	ABCA8,ACP6,AGT,ASPG,CHKA,CHKB,CLN3,DGKE,FASN,FGF7,GPAA1,HEXB,KDR,LPCAT4,LPL,NKX2- 1,PCYT2,PIGL,PIGO,PIK3CB,PIKFYVE,PIP4K2B,PISD,PLA2G4B,PLCB2,PLCG1,PLCG2,PLD2,PNPLA6,SLC27A1,SMPD1,SMPD2,SMP D3,SMPD4,TAZ	35
hydrolysis of diacylglycerol	4.52E-03	PAFAH1B1,PAFAH2,PLCG1,PLCG2	4
degradation of 2-arachidonoylglycerol	7.13E-03	FAAH,MGLL	2

Receptors, including GPCRs

Supplementary Table 2. List of overlapping F1 and F2 differentially expressed genes

Sphingolipid metabolism	
Smpd1	sphingomyelinase 1
Smpd2	sphingomyelinase 2
Smpd3	sphingomyelinase 3
Smpd4	sphingomyelinase 4
Nsmaf	neutral sphingomyelinase activation associated factor
Cerk	ceramide kinase
Asah2	ceramidase
Degs2	sphingolipid delta 4-desaturase
Fasn	fatty acid synthase
Scd	fatty acid desaturase
Cln3	neuronal ceroid-lipofuscinosis 3

Monosialodihexosylganglioside (GM3) metabolism	
UGT8	ceramide glucosyltransferase
HexB	hexosaminidase
Gm2A	GM2 ganglioside activator
Large	glucuronosyltransferase
St6galnac4	glycosyltransferase
St6galnac6	glycosyltransferase
St8sia1	glycosyltransferase

Glycerophospholipid metabolism	
Dgke	diacylglycerol kinase
Chka	ethanolamine kinase
Chkb	ethanolamine kinase
Pisd	phosphatidylserine (PS) decarboxylase
Dgat1	DAG acyltransferase1
Dgka	DAG kinase
Mgat3	monoacylglycerol acyltransferase 3
Mgll	monoglyceride lipase
Pla2g3	phospholipase A2

Inositol Phosphate Compounds	
Plcb2	phospholipase C (PLC) beta 2
Plcb4	phospholipase C (PLC) beta 4
Plcg1	phospholipase C (PLC) gamma 1
Plcg5	phospholipase C (PLC) gamma 2
Pik3cb	phosphatidylinositol (PI) kinase
Pip4k2b	phosphatidylinositol phosphate (PIP) kinase
Pip5kl1	phosphatidylinositol phosphate (PIP) kinase
Pikfyve	phosphoinositide kinase
Ppip5k1	diphosphoinositol pentakisphospate kinase 1
Ppip5k2	diphosphoinositol pentakisphospate kinase 2

Supplementary Table 3. LC/MS-based untargeted metabolite profiling of GCs

Mass	RT	Compound Name	P-value	Fold Change	WT1	WT2	WT3	WT4	WT5	WT6	F1	F2	F3	F4	F5	F6
805.555142	1.315	Lactosylceramide (d18:1/12:0)	0.001025	**0.69**	13517	18559	15358	14822	13922	12009	11975	9750	10421	9637	8659	10040
612.523	1.15	612.5229@1.153	0.001568	**0.5**	6890.6735	5870.279	8942.492	4751.243	6024.913	7343.214	4593.72	3594.054	3083.091	4511.239	2788.455	1285.39
105.041	8.48	L-SERINE	0.002494	**0.86**	545548.43	587575.4	509347.5	521087.4	519365.1	574056.4	442161.9	411167.2	501405.5	482729.2	459559.8	495996.5
480.2392	1.0579998	unknown	0.006169	**0.58**	10783	10497	7867	9628		13051	6008	6630	5628	7343	8297	2104
801.5451	1.3380002	Lactosylceramide (d18:1/12:2)	0.008609	**0.86**	27496	32597	29496	29890	30061	32112	25241	21882	29258	28627	25620	24868
815.636	1.0200001	unknown	0.008859	**0.11**	16202	14977	20811	305	3882	16565	845	1522	1422	312	423	3357
798.618	1.03	PA(43:2)	0.016535	**0.1**	37769.091	52442.19	27925.48	4068.157	1216.652	33153.52	1417.756	1980.147	3674.196	95.3798	1690.984	6445.085
789.567	7.07	PC(P-38:6)	0.017049	**0.89**	122285.75	140459.7	143802.6	123432.4	125386	135811.7	104296	121513.2	128133.7	120223.3	118336.7	109796.8
803.5448	1.3380002	Lactosylceramide (d18:1/12:1)	0.018018	**0.81**	33854	43909	34323	35551	31237	32581	26588	27149	34666	31336	26588	24662
428.364	9.98	428.3637@9.973	0.026209	**1.1**	24399.941	23841.34	24728.2	22941.06	25642.19	21499.89	24054.94	27151.02	27890.5	27945.03	24366.23	26039.64
394.2711	1.1400001	unknown	0.029537	**0.77**	88244	83537	81142	123530	99894	112448	86668	80670	62641	59516	68930	93978
322.197	1.42	322.198@1.4279999	0.03479	**1.22**	76005.85	49532.1	50517.36	67714.51	55098.65	45310.54	77237.3	71048.51	71473.84	64304.92	66765.49	69545.95
334.063	7.04	3-Phosphoglyceroinositol	0.036676	**0.81**	145326.88	154173.3	161708	121876.6	140880.5	138160.1	85926.69	107675.1	100675	127789.5	127333.5	151891.4
164.055	1.5	L-RHAMNOSE	0.043106	**1.26**	54934.681	47735.53	43796.99	45206.76	43313.29	21780.19	61212.03	56025.19	54001.76	52133.35	51993.01	48962.68
537.326	8.75	537.3266@8.748	0.044886	**1.03**	209433.44	209559.7	213559.2	218249.7	217456.3	209986.3	219850.1	213039.2	218410.3	215239	229806.5	221948.9
148.035	7.49	Methylmalate?	0.046224	**1.69**	238616.71	200796.6	226369.7	230282.5	256008.5	288905	223375	582056.7	273894.6	588074.9	527560.7	243984.3
749.5342	4.3229995	PE(P-38:5)	0.046778	**0.92**	606099	665298	745203	607464	659355	703771	568651	624384	593099	634252	624278	611696
705.528	7.74	705.5278@7.7409997	0.047103	**1.11**	343946.15	355845.8	375849.5	415715.8	376188.3	384007.2	438353.8	462037.8	441811.2	408325.7	392708.7	358888.6
666.5191	1.0130001	DG(40:7)	0.048597	**0.64**	6735	6754	11698	5242	5752	8225	4978	3262	2436	5224	7075	5612
664.5067	1.0130001	DG(40:8)	0.04449	**0.72**	9100	9391	14301	8087	10119	14045	7995	7990	4283	8710	8942	9048
311.236	1.25	311.2383@1.269	0.049318	**1.34**	23188.898	19393.18	26637.56	27256.28	20932.58	14530.15	25061.86	26581.5	41815.93	23363.96	28941.9	30960.58

PA: phosphatidic acid; PC: phosphatidylcholine; PE: phosphatidylethanolamine; DG: diacylglycerol
Statistically significant increase (fold change vs WT)
Decrease
Unpaired t-tests ($p<0.05$)

PA: phosphatidic acid
PC: phosphatidylcholine
PE: phosphatidylethanolamine
DG: diacylglycerol

Supplementary Table 4. Lipidomics data complementing Fig. 4e.

Species	F1/WT	F2/WT		Species	F1/WT	F2/WT		Species	F1/WT	F2/WT		Species	F1/WT	F2/WT
PA 36:1	1.0	0.6		PEp 36:0	2.3	1.2		PG 34:0	**0.8**	**0.8**		BMP 34:1	1.0	**0.7**
PA 36:2	0.9	0.6		PEp 36:1	**0.8**	**0.7**		PG 34:1	**0.7**	**0.8**		BMP 34:2	0.8	0.7
PA 36:3	0.8	0.6		PEp 36:2	1.2	0.9		PG 34:2	**0.7**	0.8		BMP 36:0	1.0	0.8
PA 36:4	0.8	**0.6**		PEp 36:3	**0.6**	**0.7**		PG 36:0	0.6	**0.5**		BMP 36:1	1.0	**0.6**
PA 38:0	0.9	**0.6**		PEp 36:4	1.0	1.2		PG 36:1	**0.7**	**0.6**		BMP 36:2	0.9	**0.7**
PA 38:1	0.8	**0.5**		PEp 38:0	1.2	1.0		PG 36:2	0.6	0.6		BMP 36:4	1.0	1.0
PA 38:2	0.9	**0.5**		PEp 38:1	0.8	0.7		PG 36:3	0.7	0.7		BMP 38:0	1.0	1.1
PA 38:3	0.8	**0.6**		PEp 38:2	1.0	**0.6**		PG 36:4	0.9	1.1		BMP 38:2	1.0	**0.6**
PA 38:4	0.9	**0.5**		PEp 38:3	1.1	1.1						BMP 38:3	0.8	**0.6**
PA 38:5	0.9	**0.5**		PEp 38:4	0.9	1.1						BMP 38:4	0.9	**0.7**
PA 38:6	1.0	0.7		PEp 38:5	0.9	1.1						BMP 38:6	0.9	0.9
PA 40:4	1.1	**0.5**		PEp 38:6	1.0	1.2						BMP 40:4	1.0	**0.6**
PA 40:5	1.0	**0.5**										BMP 40:5	1.0	0.8
PA 40:6	1.0	**0.5**										BMP 40:6	1.0	**0.6**
PA 40:7	1.1	**0.4**										BMP 40:7	1.0	1.0
PA 42:5	1.1	**0.5**												
**Species**	**F1/WT**	**F2/WT**		**Species**	**F1/WT**	**F2/WT**		**Species**	**F1/WT**	**F2/WT**		**Species**	**F1/WT**	**F2/WT**
LPI 16:0	**1.4**	1.1		SM d18:1/20:0	1.0	**0.8**		GalCer d18:1/20:0	**1.4**	0.9		GM3 d18:1/18:0	**5.6**	**1.7**
LPI 18:0	4.0	**1.4**		SM d18:1/20:1	0.9	0.9		GalCer d18:1/20:1	**1.5**	1.0		GM3 d18:1/20:0	**3.8**	1.8
LPI 18:1	1.1	1.2		SM d18:1/22:0	1.0	**0.8**		GalCer d18:1/22:0	1.2	0.9		GM3 d18:1/24:0	1.8	2.1
LPI 20:4	0.9	**1.3**		SM d18:1/22:1	1.0	0.7		GalCer d18:1/22:1	1.3	1.0				
				SM d18:1/24:0	1.1	0.9								
				SM d18:1/24:1	1.0	**0.8**								
				SM d18:1/26:0	1.0	0.8								
				SM d18:1/26:1	1.0	**0.7**								

Statistically significant increase (fold change vs WT)
Decrease
ANOVA, Bonferroni $p<0.05$.

PA: phosphatidic acid
PEp:plasmalogen phosphatidylethanolamine
PG: phosphatidylglycerol
BMP: bis(monoacylglycerol)phosphate
LPI: lysophosphatidylinositol
SM: sphingomyelin
GalCer: galactoceramide
GM3: monosialodihexosylganglioside

Supplementary Table 5. Functions enriched in DMR genes present in both F1 and F2 neurons

Functional Annotation	p-Value	Molecules
**Behavior**		
anxiety	2.01E-05	ADORA2A,CAMK2A,CRHR1,DRD2,GRIN3B,LMTK3,NFATC2,NPAS3,OPRD1,PCSK1N,RAI1,SHANK1,SLC6A4
hypoactivity of mice	6.89E-05	ADORA2A,DLG4,DRD2,GNAS,GRIN3B,HCN2,NR4A2,SHANK1,SLC6A4,SOCS7,SRGAP3
prepulse inhibition	1.60E-04	ADORA2A,DLG4,DRD2,GNAS,NTSR1,SLC6A4,SRGAP3
social exploration	5.03E-04	DLG4,GRIN3B,NPAS3,SLC6A4,SRGAP3
hypothermia	7.55E-04	DRD2,GALP,NTSR1,PPARA,SLC6A4
emotional behavior	1.66E-03	ADORA2A,CAMK2A,CRHR1,DLG4,DRD2,GALP,LMTK3,NPAS3,NR4A2,RIMS2,SHANK1,SLC6A4
**Lipid Metabolism**		
phosphorylation of sphingolipid	6.71E-04	CERK,SPHK1
concentration of lipid	1.67E-03	ABHD5,ADORA2A,BRD2,CEBPA,CERK,CRHR1,CXCL14,CYP26B1,DGKE,DLG4,DLK1,DRD2,GNAS,LTC4S,MBP,mir-33,NGFR,PLA2G1B,PPARA,RAI1,SIRT4,SLC5A10,SLC6A4,SMARCD3,SMPD3,SPHK1,SREBF2,SRGAP3,ST8SIA1
synthesis of ceramide	3.84E-03	CERK,DRD2,NGFR,SMPD3,SPHK1
synthesis of lipid	3.93E-03	ABHD5,ACADVL,BRD2,CEBPA,CERK,CRHR1,DGKE,DRD2,EDNRB,ITGA6,LTC4S,MAP3K1,NFATC2,NGFR,NR4A2,PDGFB,PIGY,PLA2G1B,PPARA,SIRT4,SLC22A2,SMPD3,SPHK1,SREBF2,ST8SIA1
synthesis of glycolipid	4.41E-03	CERK,DRD2,NGFR,PIGY,SMPD3,SPHK1,ST8SIA1
synthesis of glycosphingolipid	4.69E-03	CERK,DRD2,NGFR,SMPD3,SPHK1,ST8SIA1
**Neurotransmission, synapse/neuronal development**		
neurotransmission of axons	6.71E-04	EPHB2,RIMS2
proliferation of neuronal cells	1.05E-03	ADORA2A,BRSK1,CAMK2A,DLG4,DRD2,DVL2,EML1,EPHB2,GNAS,ITGA6,KIF26A,MID1,MYLIP,NFATC2,NGFR,OPRD1,PCSK1N,PDGFB,PXN,SMARCD3,SRGAP3,TIAM1,ZNF423
metabolism of dopamine	1.15E-03	DRD2,NR4A2,SLC6A4,SNCB
morphogenesis of neurites	1.87E-03	ADORA2A,ATXN10,BRSK1,CAMK2A,CRHR1,CUL7,CUX2,DLG4,DRD2,EPHB2,FGF13,MID1,NGFR,PTPN20B,SDK2,TIAM1,USP21
neurotransmission	1.98E-03	ADORA2A,CAMK2A,CRHR1,DLG4,DRD2,EPHB2,HCN2,MBP,MYH14,NGFR,NTSR1,RIMS2,RXRB,SCN8A,SLC6A4,SNCB,SNPH
neuritogenesis	2.05E-03	ADORA2A,ATXN10,BRSK1,CAMK2A,CRHR1,CUL7,CUX2,DLG4,DRD2,EPHB2,FGF13,MBP,MID1,NGFR,PTPN20B,PXN,SDK2,SNCB,ST8SIA1,STMN1,TIAM,USP21
abnormal pruning of axons	2.19E-03	EPHB2,NGFR
transport of monoamines	2.74E-03	DRD2,SLC22A2,SLC6A4
plasticity of synapse	2.75E-03	ADORA2A,CAMK2A,CRHR1,DLG4,DRD2,EPHB2,NGFR
size of dendritic spines	4.52E-03	CAMK2A,DLG4

**Supplementary Table 6**. Functions enriched in F1-F2 differentially expressed and differentially methylated genes			
Functional Annotation	p-Value	Molecules	# Molecules
Lipid Metabolism			
synthesis of glycosphingolipid	3.89E-04	CERK,SLC27A1,SMPD3,ST8SIA1	4
quantity of diacylglycerol	6.97E-04	DGKE,FAAH,SLC27A1	3
synthesis of lipid	9.22E-04	ACADVL,CERK,CRHR1,DGKE,EDNRB,FAAH,SIRT4,SLC27A1,SMPD3,ST8SIA1	10
concentration of arachidonic acid	2.42E-03	CERK,DGKE	2
hydrolysis of 1,2-dioctanoyl-sn-glycerol	6.66E-03	PAFAH2	1
phosphorylation of ceramide to ceramide-1-P	6.66E-03	CERK	1
synthesis of ganglioside GD1b,GD2,GT1,GD3	9.97E-03	ST8SIA1	1
catabolism of sphingomyelin	1.98E-02	SMPD3	1

**Supplementary Table 7**. Functions enriched in PGC differentially methylated genes			
Functional Annotation	p-Value	Molecules	# Molecules
Imprinting	3.37E-04	Blcap,Nnat, Airn/Igfr2, Ccdc40, Dio3, Wt1	5
Fertility	4.16E-04	Airn/Igfr2, Dio, Cnaz, Lhcgr, Parp1, Sulf2, Wt	7
Morphogenesis of genital organ	2.58E-03	Lhcgr, Wt	2
